# Early-life stress and ovarian hormones alter transcriptional regulation in the nucleus accumbens resulting in sex-specific responses to cocaine

**DOI:** 10.1016/j.celrep.2023.113187

**Published:** 2023-09-29

**Authors:** Devin Rocks, Ivana Jaric, Fabio Bellia, Heining Cham, John M. Greally, Masako Suzuki, Marija Kundakovic

**Affiliations:** 1 Department of Biological Sciences, Fordham University, Bronx, NY, USA; 2 Department of Psychology, Fordham University, Bronx, NY, USA; 3 Center for Epigenomics, Department of Genetics, Albert Einstein College of Medicine, Bronx, NY, USA; 4 Department of Nutrition, Texas A&M University, College Station, TX, USA; 5 Present address: Animal Welfare Division, Vetsuisse Faculty, University of Bern, Bern, Switzerland; 6 Lead contact

## Abstract

Early-life stress and ovarian hormones contribute to increased female vulnerability to cocaine addiction. Here, we reveal molecular substrates in the reward area, the nucleus accumbens, through which these female-specific factors affect immediate and conditioning responses to cocaine. We find shared involvement of X chromosome inactivation-related and estrogen signaling-related gene regulation in enhanced conditioning responses following early-life stress and during the low-estrogenic state in females. Low-estrogenic females respond to acute cocaine by opening neuronal chromatin enriched for the sites of ΔFosB, a transcription factor implicated in chronic cocaine response and addiction. Conversely, high-estrogenic females respond to cocaine by preferential chromatin closing, providing a mechanism for limiting cocaine-driven chromatin and synaptic plasticity. We find that physiological estrogen withdrawal, early-life stress, and absence of one X chromosome all nullify the protective effect of a high-estrogenic state on cocaine conditioning in females. Our findings offer a molecular framework to enable understanding of sex-specific neuronal mechanisms underlying cocaine use disorder.

## In brief

Rocks et al. reveal transcriptional mechanisms through which female-specific factors, early-life stress and ovarian hormones, induce sex-specific cocaine responses. Both factors alter X chromosome inactivation-related and estrogen signaling-related gene regulation in the nucleus accumbens. Cocaine acutely enhances ΔFosB-site accessibility, especially in low-estrogenic females, providing a ‘‘priming’’ mechanism for future exposures.

## Graphical abstract

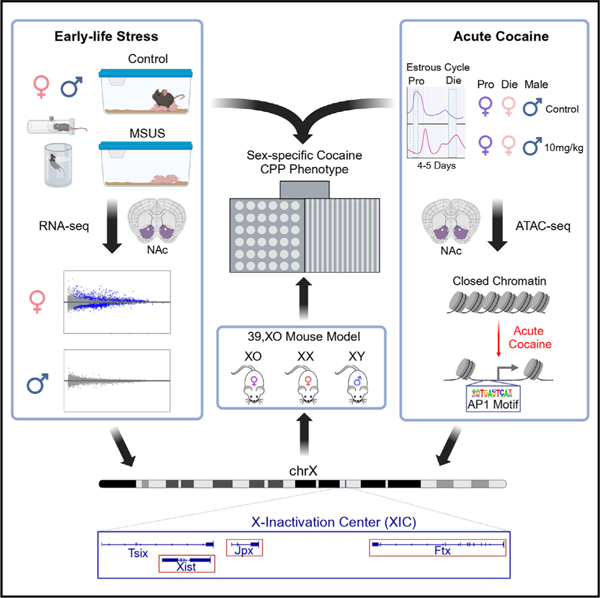

## INTRODUCTION

Substance use disorders affect people across genders, although women and men develop these disorders differently. Specifically, cocaine use disorder (CUD) affects ~1 million individuals over the age of 12 years in the United States alone,^1^ with overwhelming evidence that females are more sensitive to cocaine’s effects.^2^ Women are reported to transition to addiction faster, have more difficulty remaining abstinent, and experience more adverse consequences of cocaine use.^3^ While there is evidence that estradiol potentiates the cocaine-induced ‘‘high,’’^4,5^ very few studies have addressed the underlying molecular mechanism. In fact, animal studies have focused on males,^6^ missing the opportunity to reveal sex-specific mechanisms underlying reward processing and cocaine addiction.

Clinical studies including childhood trauma survivors offer insights into the increased female vulnerability to cocaine. Early-life trauma is a major risk factor for the development of mental disorders,^7,8^ and animal studies have linked early-life stress (ELS) to addiction-related behaviors across sexes.^9–11^ However, in humans, the association between childhood trauma and the development of substance use disorders is generally stronger in women,^12–15^ with early trauma increasing the likelihood of cocaine relapse and drug-use escalation in women but not in men.^16^ Revealing sex-specific and trauma-related pathophysiology of cocaine addiction can be beneficial in improving CUD treatment outcomes in people of all genders.

Among molecular mechanisms, chromatin and gene expression changes in the key brain reward area, the nucleus accumbens (NAc), have long been proposed to underlie both the acute and chronic effects of cocaine.^17,18^ However, the sex specificity of cocaine’s effects is understudied. We recently showed that neuronal chromatin organization, a major mechanism controlling gene expression, changes in the ventral hippocampus with sex and estrous cycle stage.^19^ Earlier studies have also shown transcriptional and chromatin changes in the NAc in response to ELS.^20–22^ Therefore, we hypothesized that ELS and ovarian hormone status can affect transcriptional and chromatin regulation in the NAc, leading to sex-specific responses to cocaine.

Here, we designed a comprehensive mouse study to address sex difference in cocaine sensitivity at both the etiological and mechanistic level. We first demonstrate the effects of the two major sex-specific risk factors in the etiology of CUD, ELS and ovarian hormone status, on the acquisition of cocaine preference in mice. We then characterize the NAc transcriptome and epigenome to reveal both shared and distinct transcriptional mechanisms by which these two factors drive sex-specific responses to cocaine, including an unexpected involvement of the inactive X chromosome (Xi), which we functionally linked to cocaine-induced behavior.

## RESULTS

### ELS study design

**Figure 1.**
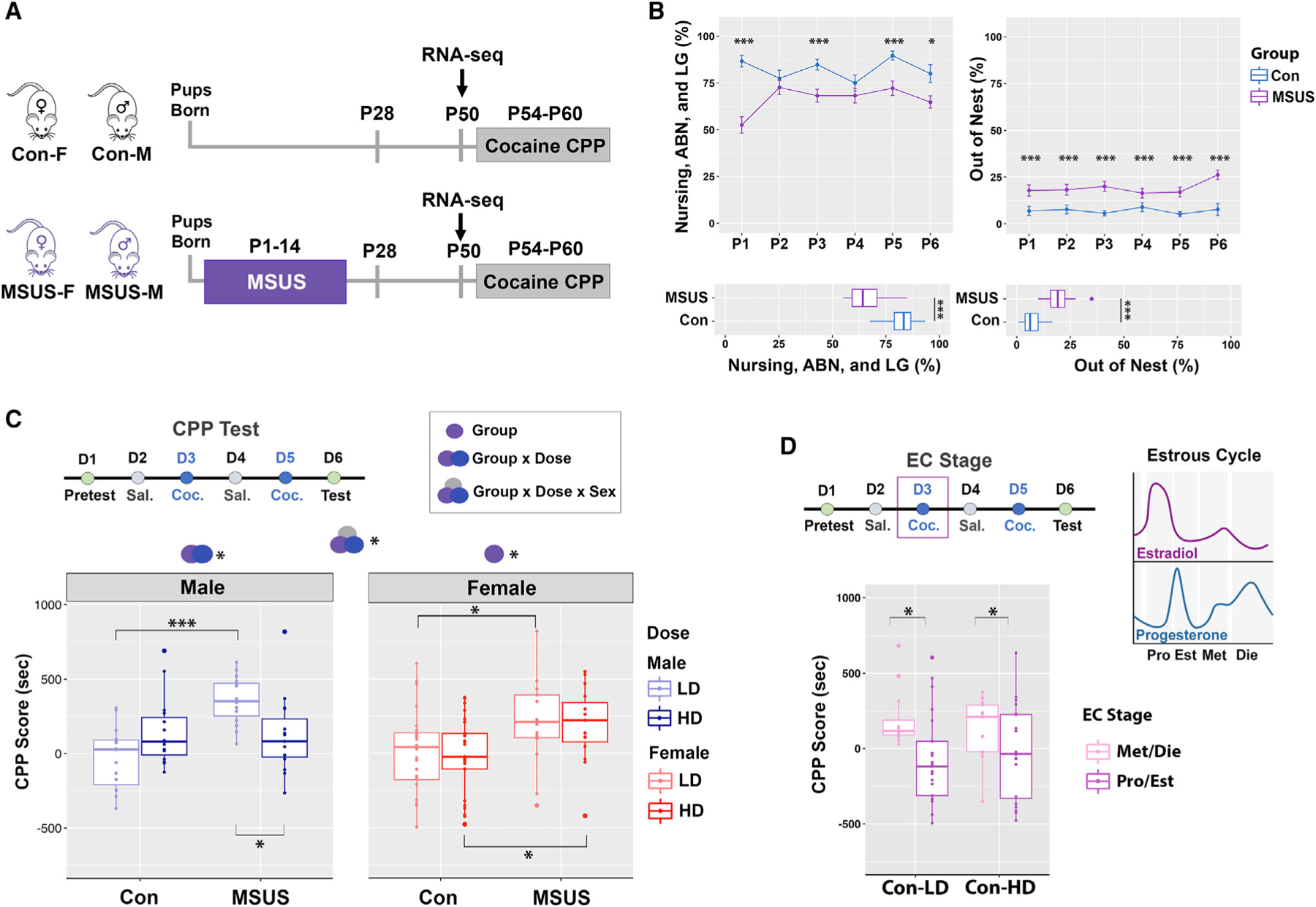
ELS alters cocaine preference in a sex- and dose-dependent manner (A) ELS study design included Con (control) and MSUS (maternal separation with maternal unpredictable stress, from postnatal [P] day 1–14) groups. (B) Maternal behavior tracking following separation included the time spent nursing, arched-back nursing (ABN), and licking/grooming (LG) across P1–P6 (upper left) and on average (lower left); and the time spent out of the nest across P1–P6 (upper right) and on average (lower right). Repeated-measures ANOVA with the Tukey’s post hoc test (upper plots; error bars, standard error); Welch’s two-sample t test (lower plots). (C and D) In the cocaine CPP test, scores were analyzed with group (Con/MSUS), sex (male/female), and dose (high/low) as factors (C, n = 15–25 animals/sex/dose/group) or, separately in control females, with dose and estrous cycle (EC) stage as factors (D, n = 12–23/stage/dose). Symbols above the graphs show significant main effects of group, sex, dose, or their interaction. Three-way ANOVA (group × dose × sex interaction); two-way ANOVA (male, female, and estrous cycle plots). Box plots: box, 1st–3rd quartile; horizontal line, median; whiskers, 1.5× interquartile range (IQR). F, female; M, male; D, day; Sal., saline; Coc., cocaine; LD, low dose; HD, high dose; Pro, proestrus; Est, estrus; Met, metestrus; Die, diestrus. *p < 0.05; ***p < 0.001.

To determine the effects of ELS on addiction-related behavioral and molecular phenotypes, we used a previously established ELS paradigm.^23–25^ Mouse litters were assigned to either the control (Con) group that received no stress or an ELS group that underwent a 3-h maternal separation combined with maternal unpredictable stress (MSUS) daily from postnatal days 1–14 (P1–P14) (Figure 1A, see STAR Methods). We also monitored maternal behaviors during the first 6 days postpartum (P1–P6), a period critical for mother-infant interactions in mice,^26^ and confirmed that there was a disruption in maternal care for at least 1 h following separation (Figure 1B). Specifically, we found reduced nurturing behaviors including nursing, arched-back nursing, and licking/grooming, as well as increased time spent out of the nest, in MSUS compared to control mothers across the 6 days and on average (Figure 1B). Animals were weaned at P28 and underwent behavioral testing using the cocaine-induced conditioned place preference (CPP) test during the late adolescent period in mice (P54–P60; Figure 1A). A subset of Con and MSUS mice were sacrificed at P50, just prior to the onset of CPP, and used for gene expression (RNA sequencing [RNA-seq]) analysis of the NAc (Figure 1A).

### ELS alters cocaine preference in a sex- and dose-dependent manner

To evaluate the effects of ELS on cocaine preference, we performed the CPP test using two cocaine doses: the higher dose (HD, 10 mg/kg intraperitoneally [i.p.]), which effectively induces cocaine preference,^27^ and the lower dose (LD, 2.5 mg/kg i.p.), considered insufficient to induce CPP under basal conditions.^27,28^ HD or LD cocaine was administered to mice of both sexes in each group on conditioning days (days [D] 3 and 5, Figure 1C). To account for estrous cycle effects on CPP score in females, we performed estrous cycle tracking daily across the experiment (D1–6, Figure S1A).

We first analyzed the CPP score using a three-way ANOVA with experimental group (Con or MSUS), sex (female or male), and dose (HD or LD) as factors, revealing a significant group-by-sex-by-dose interaction (Figure 1C and Table S1A). To further understand this three-way effect, we analyzed the effect of group and dose, and their interaction, separately in each sex (Table S1A). Within males, we found that the effect of ELS depends on dose (Figure 1C). The high cocaine dose induced a preference for the cocaine-paired compartment in both control (Con-HD) and MSUS (MSUS-HD) male groups, with no significant difference between these two groups (Figure 1C). However, ELS had a significant effect with the lower dose of cocaine (Table S1A), where MSUS males (MSUS-LD) showed cocaine preference that was not observed in control males (Con-LD). Interestingly, within MSUS males, the LD group had a higher CPP score than the HD group, indicating that ELS reversed the expected dose-response relationship in male animals (Figure 1C).

Within females we observed a significant effect of group, with no effect of dose and no group-by-dose interaction (Figure 1C and Table S1A). Further analysis within females revealed a significant main effect of group in both the LD and HD groups, with MSUS females exhibiting higher cocaine-induced preference than control females regardless of cocaine dose.

Notably, unlike males, control females receiving the high cocaine dose (Con-HD) showed no overall preference for the cocaine-paired compartment, with an average CPP score of −17.50 (±256.23) and individual responses that varied from preference to aversion (Figure 1C). Considering that estrogen modulates cocaine’s effect on the brain reward system,^2^ we hypothesized that the varied CPP score in control females may be explained by their ovarian hormone status. Importantly, the CPP paradigm lasts 6 days, and hormone status on any of these days (D1–6) could influence animal performance during the test (D6). We noticed that cocaine may affect estrous cycling, consistent with previous literature,^29^ so for our analysis we focused on the animals’ estrous cycle stage on their first exposure to cocaine (D3, Figure 1D). To increase the power of the analysis, females were classified into two groups: (1) Pro/Est animals (occupying proestrus, estrus, or transitionary phases) with higher levels of estradiol; and (2) Met/Die animals (occupying metestrus, diestrus, or transitionary phases) with lower levels of estradiol (Figure S1B). We analyzed control female CPP data using two-way ANOVA with estrous cycle stage (Pro/Est or Met/Die) and dose (HD or LD) as factors (Figure 1D and Table S1B). We initially found no effect of the estrous cycle, dose, or their interaction (Table S1B). After dropping the interaction effect, however, we found a significant effect of the estrous cycle stage, with Met/Die control females exhibiting higher CPP scores than control Pro/Est females across the two doses (Figure 1D). These data indicated that high estrogen levels in the Pro/Est group may be a protective factor against the conditioning effects of cocaine (Figure 1D), and this effect seems to be lost following ELS (Figure 1C).

Overall, we found that our ELS paradigm increases cocaine preference in late adolescence in both male and female mice. However, the ELS effect is stronger in females, increasing cocaine preference across the two doses while having an effect with only the low cocaine dose in males.

### Transcription in the NAc is altered by ELS in a sex-specific manner

**Figure 2.**
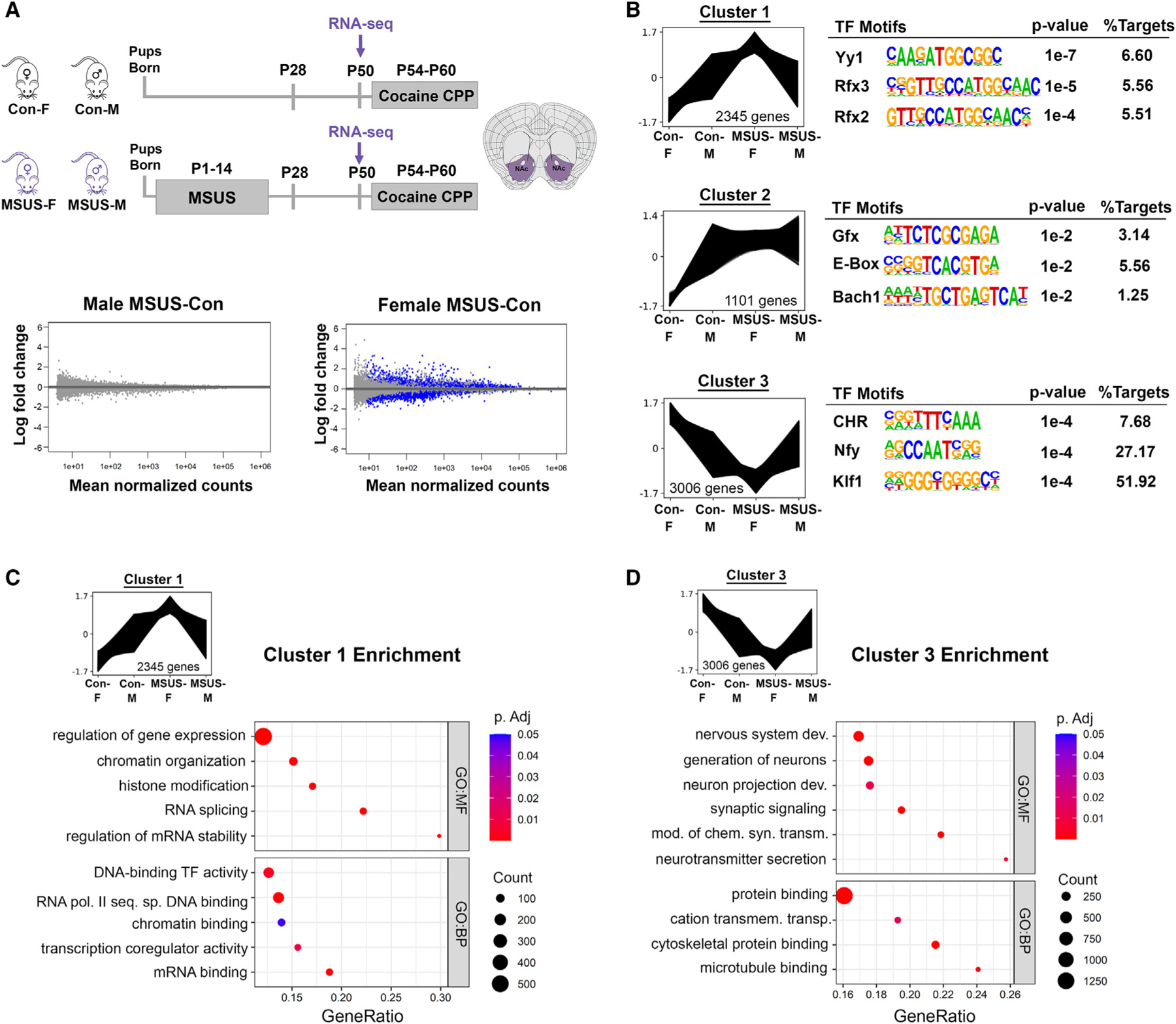
ELS results in sex-specific changes in NAc gene expression (A) RNA-seq was performed on the NAc from female and male mice in Con and MSUS groups (n = 6 animals/sex/group). Volcano plots depict differentially expressed genes across groups within males (n = 0) and females (n = 5,527); blue dots represent significant genes (p_adj_ < 0.1, DESeq2). (B) Clust identified three significant gene clusters across the four experimental conditions (left) shown with corresponding Homer-identified top-enriched binding motifs in promoter regions of cluster genes (right). (C and D) Dot plots depict select gene ontology (GO) terms for biological process (BP) and molecular function (MF) significantly enriched for cluster 1 (C) and cluster 3 (D) genes. Colors indicate adjusted p values, and dot size corresponds to gene count. Con, control; MSUS, maternal separation with unpredictable maternal stress; F, female; M, male.

To determine the transcriptional signatures of ELS that preceded the observed cocaine-induced behavioral phenotypes, we performed RNA-seq on the NAc of MSUS and Con males and females at P50 (n = 6/group/sex; Figure 2A), ensuring that Con and MSUS female groups have a balanced number of high- and low-estrogenic animals (see STAR Methods). We first compared control males and females and found 1,508 genes (p_adj_ < 0.10) with sex-specific expression in the NAc, including genes relevant for the regulation of chromatin and transcription, response to stress, and synaptic function (Figure S2). Importantly, the number of sex-specific genes decreased to 163 following ELS (p_adj_ < 0.10; Figure S2), implying that ELS reduces sex differences in the NAc, consistent with the CPP phenotype that becomes more similar between males and females after ELS (Figure 1C and Table S1).

We then performed differential gene expression analysis between MSUS and Con in each sex and separately tested for group-by-sex interaction (Figures 2A and S3). Remarkably, this analysis revealed 5,527 differentially expressed genes (DEGs) between MSUS and Con within females, while no DEGs were identified in males using a p_adj_ < 0.10 cutoff. A total of 455 DEGs were identified as having a significant group-by-sex interaction; these genes were enriched for metabolic and brain-disease-relevant terms (Figure S3). Next, we compared our dataset to that of two other studies that performed RNA-seq on the NAc of male and female mice exposed to ELS^20,21^ (Figure S4). Although our three studies used different ELS paradigms, the common finding was that ELS affects female gene expression more profoundly, with our MSUS paradigm showing the most extreme sex difference. Specifically, when we used the looser cutoff criteria that corresponded to the other two studies,^20,21^ we found 15 female DEGs that overlapped between the three paradigms, showing enrichment relevant to neuronal development and neurogenesis (Figures S4A and S4B). Using the same criteria, in males we identified a similar number of DEGs as in the other two studies, but no genes overlapped between the three datasets (Figure S4A), indicating subtler and less consistent changes in male NAc gene expression following ELS. Interestingly, in the overlap between our female data with the female data derived from the ELS study combining maternal separation and limited bedding (P10–P17),^20^ we found genes enriched for addiction-relevant pathways (Figure S4C). Overlapping our female data with that of the limited bedding and nesting (P2–P10) paradigm,^21^ however, revealed altered glutamatergic-relevant, transcription-relevant, and reproductive-axis-relevant genes (Figure S4D).

To include both females and males in our analysis of the RNA-seq data and address sex differences, given that we did not identify DEGs in males at the p_adj_ < 0.1 threshold, we leveraged analysis methods that consider the expression profiles of all detected genes. We first performed gene co-expression clustering analysis using Clust^30^ (see STAR Methods), which identified three distinct clusters of genes with co-varying expression across the four conditions (Figure 2B and Table S2F). Cluster 1 contains 2,345 genes that, on average, are more highly expressed in male than female controls. This sex difference is reversed in the MSUS group due to upregulation of expression in females and lack of a transcriptional response in males (Figure 2B; e.g., Figure S5). Motif analysis on this cluster revealed top enrichment for binding sites of Yy1 (Figure 2B), a transcription factor implicated in stress response.^31^ Although Yy1 is predicted to regulate cluster 1 genes that are upregulated in MSUS females, Yy1 expression itself is downregulated in this group (Figure S6A and Table S2A), possibly due to its transcriptional autoregulation.^32^ Cluster 2 contains 1,101 genes with the top Gfx motif; these genes exhibit an expression pattern similar to that of cluster 1, except for a relatively reduced upregulation in MSUS females, making females more similar to males (Figure 2B; e.g., Figure S5). Cluster 3, by contrast, contains 3,006 genes that are more highly expressed in control females than control males. This sex difference is also reversed after ELS, due to downregulation in females (Figure 2B; e.g., Figure S5). Interestingly, >50% of cluster 3 genes harbor the Klf motif (Figure 2B). Several Klf transcription factors are downregulated in MSUS females (Figure S6B and Table S2A), including Klf9, which is implicated in blunting neuronal plasticity during stress exposure.^33^

We focused our subsequent analysis on clusters 1 and 3, since they contain the highest number of genes with the highest degree of change. Gene ontology (GO) enrichment analysis revealed that cluster 1 genes, upregulated in MSUS females, are broadly involved in gene regulation, chromatin organization, and RNA processing (Figure 2C), indicating that ELS induces upregulation of genes related to transcriptional control in the female NAc. In cluster 3, representing genes downregulated in MSUS females, GO enrichment analysis revealed terms related to neurotransmission and synaptic function, indicating a downregulation of genes important for neuronal function in the female NAc following ELS (Figure 2D).

### ELS alters gene expression related to estrogen signaling and dosage compensation in females

**Figure 3.**
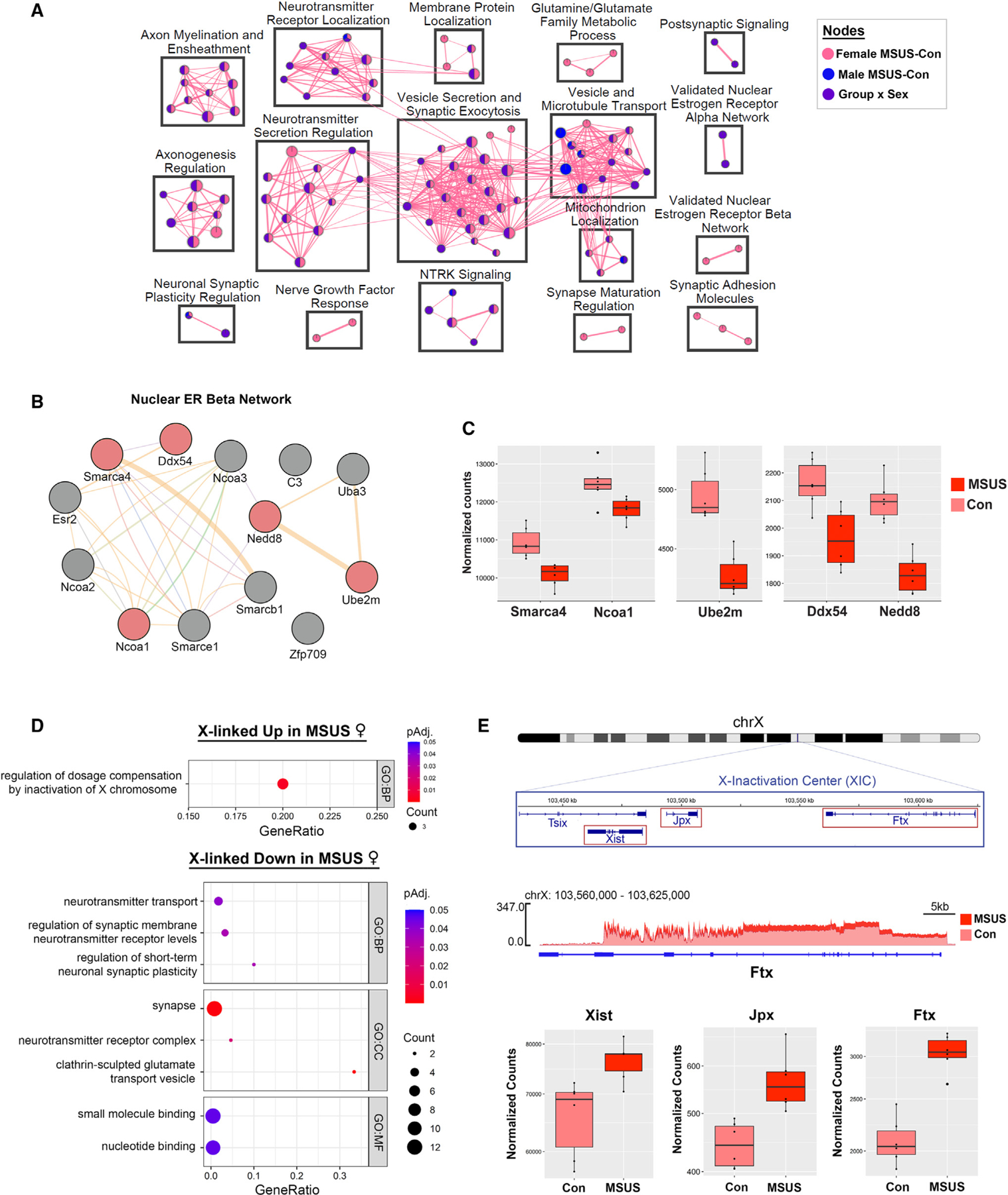
ELS affects NAc gene expression related to estrogen signaling and X chromosome regulation in females (A) Enrichment map with results for the gene set enrichment analysis. Each node in the enrichment map represents a pathway significantly enriched in one or more gene lists (denoted by node color), while edges represent genes shared between pathways. (B) The Nuclear Estrogen Receptor (ER) Beta Network pathway, significantly enriched in the female analysis, was visualized using GeneMania, with each circle representing a gene in the pathway and leading-edge genes colored green. Edges represent protein-protein interactions between pathway gene products. (C) Normalized count plots are shown for each of the leading-edge genes depicted in (B) for the female analysis, including *Smarca4* (p_adj_ = 0.0129), *Ncoa1* (p_adj_ = 0.0934), *Ube2m* (p_adj_ = 8.36e–05), *Ddx54* (p_adj_ = 0.0238), and *Nedd8* (p_adj_ = 0.00108). (D) Gene ontology (GO) analysis on X-linked genes altered in MSUS females (p_adj_ < 0.1, DESeq2) show the only term enriched in upregulated genes (top) and selected terms for biological process (BP), cellular component (CC), and molecular function (MF) enriched in downregulated genes (bottom). Colors in dot plots indicate adjusted p values, and dot size corresponds to gene count. (E) The X chromosome is shown with an inset corresponding to the X-inactivation center (XIC). *Ftx* expression in female Con and MSUS groups is shown with a SparK plot of group-average normalized RNA-seq reads across gene locus (middle, n = 6 replicates/group). Normalized count plots show expression of *Xist* (p_adj_ = 0.0226), *Jpx* (p_adj_ = 0.00163), and *Ftx* (p_adj_ = 3.31e–09) in females (bottom). Box plots: box, 1st–3rd quartile; horizontal line, median; whiskers, 1.5× IQR. Con, controls; MSUS, maternal separation with maternal unpredictable stress.

To further identify the biological pathways affected by sex and ELS in the NAc, we performed gene set enrichment analysis (GSEA^34^) on the ranked gene lists from three comparisons: MSUS vs. Con males, MSUS vs. Con females, and a group-by-sex interaction analysis (Figure 3A; see STAR Methods). GSEA revealed pathways affected by ELS in males, including Ntrk2 signaling and regulation of short-term neuronal plasticity (Figure 3A and Table S3). As expected, more pathways were identified in females and in the group-by-sex interaction analysis than in males, including pathways related to synaptic vesicle regulation, neurotransmission, and axonogenesis (Figure 3A and Table S3). Among female-specific pathways, we found that expression of genes in the nuclear estrogen receptor β (ERβ) network was affected by ELS. Given the role of ERβ in mediating cocaine’s effects in female mice,^4^ we investigated the specific genes affected and found that 5 out of 13 genes in the ERβ network exhibited altered expression after MSUS (Figure 3B), and all were downregulated in MSUS females (Figure 3C and Table S2A). A deficit in estrogen receptor signaling in the NAc of MSUS females is consistent with their increased cocaine preference, given the protective effect of high estrogen on cocaine CPP we observed in control females (Figure 1D).

Altered expression of genes related to sex hormone signaling can certainly provide a molecular basis for the sex differences in behavior we observed. However, another possible mechanism involves differences in expression of sex chromosome-linked genes between males and females. We therefore performed GO analysis of X-linked genes whose expression is altered (p_adj_ < 0.1) by ELS in females (Figure 3D). Intriguingly, X-linked genes upregulated in MSUS females were enriched for only one term: regulation of dosage compensation by inactivation of X chromosome (Figure 3D). By contrast, several terms were identified in X-linked genes downregulated by ELS, including terms related to synapse, glutamate signaling, and synaptic plasticity (Figure 3D). These results mirror those of the cluster analysis, namely the upregulation of genes involved in transcriptional control and downregulation of genes involved in neuronal function in MSUS females. Remarkably, among the X-linked genes upregulated in MSUS females were long non-coding RNAs (lncRNAs) present in the X-inactivation center^35^ that play integral roles initiating and maintaining X inactivation: Xist,^36^ Jpx,^37^ and Ftx^38^ (Figure 3E). In addition, ELS altered expression of other genes, both X-linked and autosomal, involved in X chromosome inactivation (Figure S7A) including Xist m^6^a methylation^39^ (*Rbm15*, *Rbm15b*, *Ythdc1*), Xi localization^40^ (*Firre*), and Xi chromatin repression^41,42^ (*Smchd1*, *Lrif1*). Downregulated X-linked genes in MSUS females include those involved in the glutamate receptor complex (*Porcn*,^43^ *Dlg3*,^44,45^ *Iqsec2*^46^) and synaptic vesicle regulation (*Cdk16*,^47^ *Syn1*,^48^ *Syp*,^49^ *Gdi1*^50^; Figure S7B). While we are unable to resolve the relative contributions of the active and inactive X chromosomes to the observed changes in gene expression, it is noteworthy that several genes downregulated in MSUS females have been observed to escape from X inactivation in the mouse brain,^51^ including *Eif2s3x*, *Gdi1*, and *Syp* (Table S2A).

Overall, ELS impacts the expression of genes involved in estrogen signaling and X chromosome inactivation in the female NAc, which likely contribute to the sex-specific CPP phenotype that we observed. Considering the significant X chromosome dynamics across the estrous cycle,^52^ it is plausible that the two female-specific factors, ovarian hormone status and X chromosome regulation, interact to shape sex-specific brain regulation under basal conditions and in response to environmental factors such as stress and cocaine exposure.

### Acute cocaine exposure leads to neuronal chromatin reorganization in the NAc

**Figure 4.**
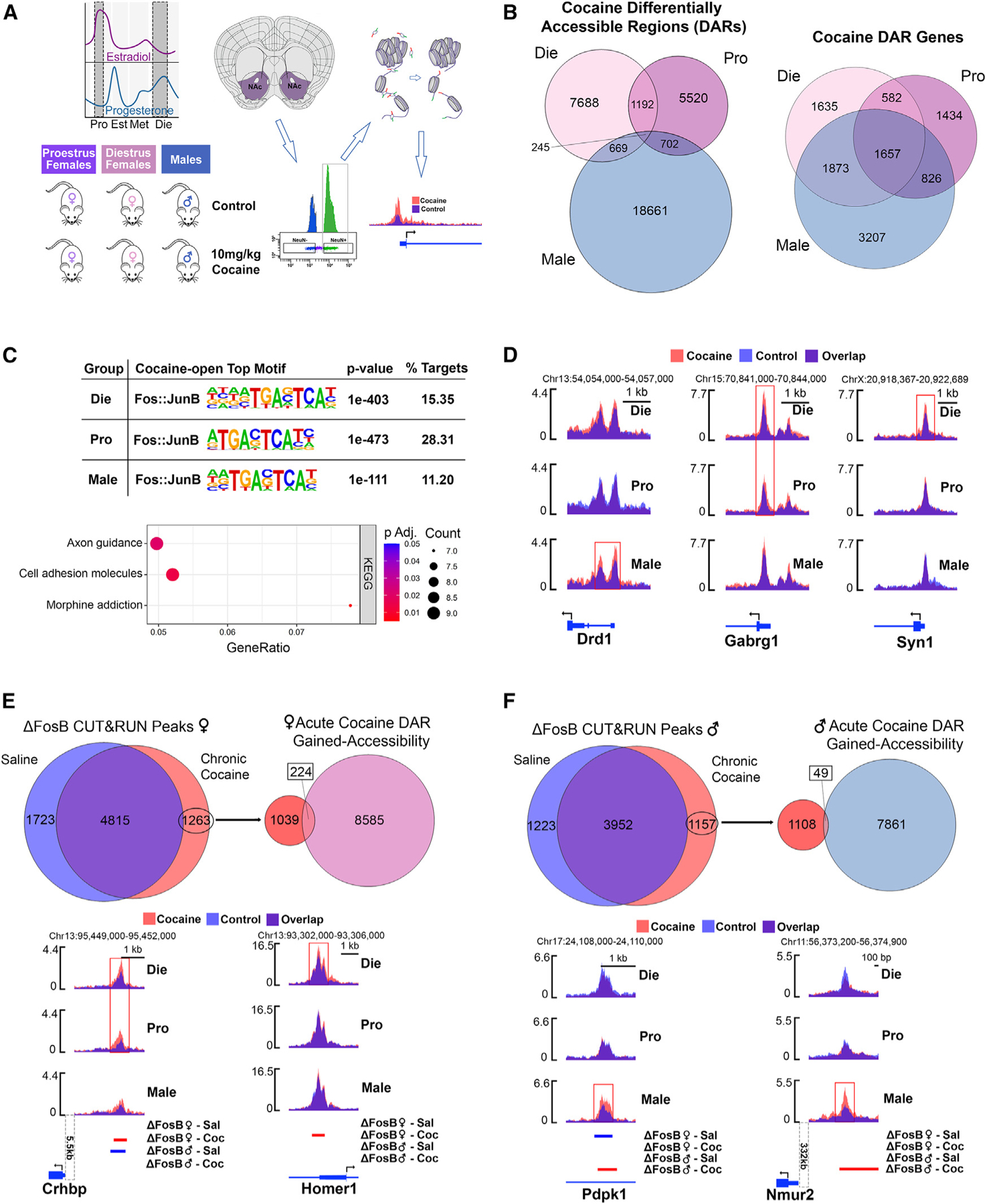
Acute cocaine exposure alters chromatin accessibility of ΔFosB binding sites in NAc neurons (A) ATAC-seq study design. Proestrus and diestrus phases of the estrous cycle are highlighted with the corresponding estradiol and progesterone levels. (B) Venn diagrams show overlaps of cocaine-induced differentially accessible regions (DARs) (left) and the corresponding genes (right) across diestrus, proestrus, and male groups. (C) Regions that gain accessibility after acute cocaine are enriched for the Fos-JunB (AP1) motif as the top motif in each group (top), and a dot plot shows the top KEGG pathways enriched in genes with cocaine-induced, AP1 motif-containing open regions in all three groups (bottom). Colors indicate adjusted p values, and dot size corresponds to gene count. (D) SparK plots are shown for sex-specific genes with gained accessibility of Fos-JunB motifs after acute cocaine, including regions near *Drd1* (left), *Gabrg1* (middle), and *Syn1* (right). Red boxes depict cocaine-induced DARs. (E and F) We then overlapped CUT&RUN data^54^ of ΔFosB binding in (E) female and (F) male NAc Drd1-expressing medium spiny neurons after chronic cocaine exposure with our ATAC-seq data on NAc neurons from females and males after acute cocaine treatment, respectively. For both females (E) and males (F), we focused on regions specifically bound by ΔFosB in the chronic cocaine condition (left Venn diagram) and overlapped them with regions that gain accessibility after acute cocaine (right Venn diagram). Shown below are exemplary SparK plots of overlapping genes including: (E) female-specific regions of *Crhbp* (left) and near *Homer1* (right); and (F) male-specific regions of *Pdpk1* (left) and upstream of *Nmur2* (right). Red boxes depict cocaine-induced DARs; bed tracks below depict ΔFosB binding in chronic cocaine and chronic saline conditions for males and females. SparK plots depict group-average normalized ATAC-seq reads (n = 2–3 replicates or 6–9 animals/group). Die, diestrus; Pro, proestrus; Male, males.

While the impact of ovarian hormones on cocaine’s reinforcing effect in the NAc is well established,^5^ the underlying molecular mechanisms are underexplored. Our CPP data indicated that the estrous cycle state at the first cocaine exposure affects the acquisition of cocaine preference, making the female cocaine response more variable than that of males at 10 mg/kg dose under basal conditions (Figures 1C and 1D). We decided to explore a transcriptional mechanism in NAc neurons through which acute cocaine treatment induces the observed estrous cycle-specific and sex-specific effects, hypothesizing that cocaine would differentially affect neuronal chromatin in the NAc of males and females across the estrous cycle.^19^ For chromatin accessibility experiments we were able to include groups with better-defined ovarian hormone levels: proestrus (high-estrogen, low-progesterone) and diestrus (low-estrogen, high-progesterone) females, which mimic human follicular and luteal phases, respectively^19^ (see STAR Methods). To test our hypothesis, we performed assay for transposase-accessible chromatin with sequencing (ATAC-seq)^53^ on purified NAc neuronal nuclei from proestrus female, diestrus female, and male mice 1 h after exposure to 10 mg/kg cocaine alongside group-matched controls (Figure 4A).

Interestingly, an initial clustering analysis indicated that cocaine exposure substantially reduced sex differences in NAc chromatin organization (Figure S8A). Strikingly, when we compared the differentially accessible regions (DARs) between males and females, we found that acute cocaine exposure leads to a more than 10-fold reduction in the number of sex-specific open chromatin regions in both male-diestrus and male-proestrus comparisons (Figure S8B). We then focused on chromatin changes occurring between control and cocaine conditions separately in each group–diestrus, proestrus, and males (Figures 4B and S9; Table S4). Initially, we found minimal overlaps in specific cocaine-induced DARs between groups (Figure 4B). However, once DARs were annotated to the nearest gene, we identified a similar number of genes with cocaine-induced chromatin changes in males, diestrus females, and proestrus females, with a higher overlap between groups (Figure 4B). The highest number of genes with affected chromatin found in males (7,563) was in line with the more consistent CPP effect found in control males at this dose compared to control females (Figure 1C). Within females, proestrus mice had a lower number of genes with cocaine-induced chromatin changes (4,499) compared to diestrus mice (5,747), consistent with the finding that females in a high-estrogenic phase during their first cocaine exposure exhibit a lower conditioning response to cocaine (Figure 1D). Examining the overlapping genes between groups, we found the most substantial overlap between diestrus females and males (3,530; p < 2.2e–16, Fisher’s exact test [FET]), the two cocaine CPP-susceptible groups, compared to diestrus and proestrus (2,260; p < 2.2e–16, FET) or proestrus and males (2,483; p < 2.2e–16, FET; Figure S9A). GO and Kyoto Encyclopedia of Genes and Genomes (KEGG) enrichment analysis of the genes with cocaine-induced DARs found, overall, that similar terms and pathways are affected by cocaine in each group, including synapse and dendritic spine organization, dopaminergic synapse, and addiction-related pathways (Figure S9B).

To assess whether neuronal chromatin accessibility is associated with gene expression, we performed two sets of analyses. First, we correlated our NAc ATAC-seq and RNA-seq data from control groups and found that chromatin accessibility in gene promoter regions is significantly associated with gene expression levels (Figure S10A and Table S5), consistent with previous studies.^55^ Next, to link cocaine-induced chromatin changes to changes in gene expression, we overlapped our ATAC-seq data with previously published NAc RNA-seq data following acute, 1-h cocaine treatment in males and females.^56^ We found a significant overlap between genes with cocaine-induced chromatin changes (Table S4) and cocaine-induced DEGs^56^ in males (p < 2.2e–16, FET), proestrus females (p = 1.69e–7, FET), and diestrus females (p = 2.92e–9, FET; Figure S10B), indicating a significant association between acute changes in chromatin and gene expression in the cocaine response.

In summary, acute cocaine exposure alters neuronal chromatin accessibility in males, diestrus females, and proestrus females, with enrichment near genes related to neuronal function and addiction, overall reducing sex differences in NAc chromatin organization. Chromatin accessibility changes are significantly associated with immediate changes in NAc gene expression,^56^ although many of these chromatin changes may also ‘‘prime’’ the genome for later transcriptional responses.^57^

### Acute cocaine exposure increases the accessibility of regions harboring AP1 motifs

To identify upstream regulators of cocaine’s effects on neuronal chromatin, we performed motif analysis on DARs that are more accessible after acute cocaine exposure in each group. In all three groups, the top motif was Fos-JunB (Figure 4C), corresponding to the binding site for the AP1 transcription factor formed by dimers of Fos- and Jun-family proteins.^58^ Genes with Fos-JunB-motif-containing DARs shared by all three groups are enriched for the Morphine addiction KEGG pathway (Figure 4C), consistent with the well-established role of Fos proteins in mediating addiction-related molecular phenotypes in the NAc.^59^ However, examination of promoter DARs harboring Fos-JunB motifs found sex- and estrous cycle-specific effects of cocaine; diestrus females had the largest number of such DARs (689), followed by proestrus females (74) and males (37) (Table S6). The vast majority of Fos-JunB motif-containing DARs were sex specific, including a male-specific DAR at the promoter of *Drd1* (Figure 4D) encoding the dopamine receptor D1, extensively involved in mediating cocaine’s effects,^60^ and a female-specific DAR at the promoter of *Gabrg1* (Figure 4D) encoding GABA receptor G1, a gene whose variants in the human paralog are associated with addiction risk.^61^ Diestrus-specific promoter DARs with AP1 sites include *Syn1* (Figure 4D), an X-linked gene whose expression is altered following ELS in females (Figure S7B).

Sites harboring the Fos-JunB motif could be bound by AP1 proteins such as cFos, implicated in mediating the acute effects of cocaine,^62^ or by ΔFosB, implicated in the transcriptional effects of chronic cocaine exposure.^62–67^ We therefore tested the overlap of our regions that gain chromatin accessibility after acute cocaine exposure with regions bound by cFos^68^ or ΔFosB^54^ derived from publicly available chromatin immunoprecipitation sequencing and CUT&RUN datasets, respectively. This analysis revealed a higher ATAC-seq signal in ΔFosB-bound regions compared to cFos-bound regions, which was most evident in the diestrus group (Figure S11), indicating that cocaine acutely alters chromatin accessibility in targets of ΔFosB, priming them for regulation following chronic cocaine exposure. To further evaluate this hypothesis, we overlapped regions that gain accessibility after acute cocaine in our data with regions bound by ΔFosB in Drd1-expressing medium spiny neurons (MSNs) specifically after chronic cocaine treatment.^54^ We found that 17.7% (224/1,263; p = 0.004, MCFDR) of these regions, bound by ΔFosB in the chronic cocaine, but not saline, condition gain accessibility after acute cocaine exposure in females (Figure 4E). Of these regions, 49.6% are unique to diestrus, 25.4% are unique to proestrus, and 25% are shared between the two female groups (Table S6). Examples include a region upstream of *Crhbp*, encoding corticotropin-releasing hormone-binding protein, involved in addiction^69,70^ and sex differences in stress response.^71^ This *Crhbp* region gains accessibility after acute cocaine and gains ΔFosB binding after chronic cocaine specifically in females (Figure 4E) while losing ΔFosB binding after chronic cocaine treatment in males (Figure 4E). The promoter region of *Homer1*, encoding a transcription factor involved in cocaine-induced synaptic plasticity,^72^ similarly gains ΔFosB binding after chronic cocaine only in females, but only gains chromatin accessibility after acute cocaine in diestrus females (Figure 4E). Notably, in males only 4.2% (49/1,157; p = 0.004, MCFDR) of regions gaining ΔFosB binding after chronic cocaine overlap with a region that gains accessibility after acute cocaine exposure (Figure 4F). These regions include an intronic region of *Pdpk1*, encoding 3-phosphoinositide-dependent protein kinase-1, and a region upstream of *Nmur2* (Figure 4F), encoding a neuropeptide receptor whose NAc expression is dysregulated following repeated cocaine administration.^73,74^

Together, these data show that acute cocaine exposure sex-specifically increases chromatin accessibility in AP1-motif-containing regions that exhibit sex-specific ΔFosB binding after chronic cocaine treatment. Notably, diestrus females had the greatest number of overlaps, and gained accessibility of ΔFosB binding sites therefore represents a putative epigenomic mechanism through which ovarian hormone status during acute exposure to cocaine can affect susceptibility to future exposures.

### Cocaine’s effects on chromatin accessibility are sex dependent and estrous cycle stage dependent

**Figure 5.**
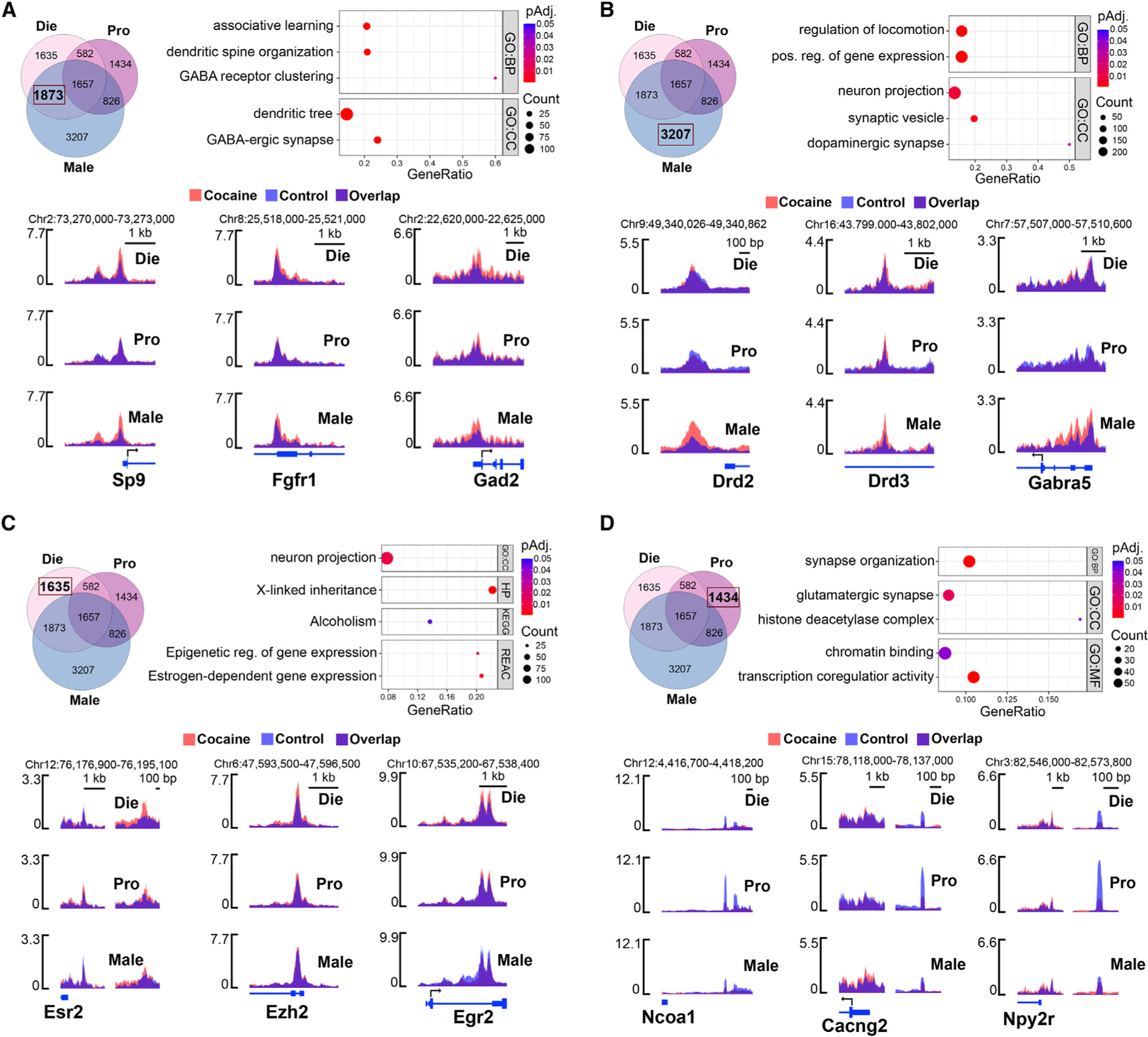
Acute cocaine alters chromatin accessibility in a sex- and estrous cycle stage-specific manner (A–D) For each panel, Venn diagrams (left) show overlapping genes corresponding to cocaine-induced differentially accessible regions (DARs) across groups, while dot plots (right) show select terms enriched in genes that are: (A) shared by males and diestrus females, but not proestrus females; (B) male specific; (C) diestrus specific; and (D) proestrus specific. SparK plots are shown below for: (A) male-diestrus overlapping genes that gain accessibility including *Sp9* (left), *Fgfr1* (middle), and *GAD2* (right); (B) male-specific genes that gain accessibility including *Drd2* (left), *Drd3* (middle), and *Gabra5* (right); (C) diestrus-specific genes that gain accessibility including *Esr2* (left), *Ezh2* (middle), and *Egr2* (right); (D) proestrus-specific genes that lose accessibility including *Ncoa1* (left), *Cacng2* (middle), and *Npy2r* (right). In dot plots, colors indicate adjusted p values, and dot size corresponds to gene count. Die (light pink), diestrus; Pro (purple), proestrus; Male (blue), males. SparK plots depict group-average normalized ATAC-seq reads (n = 2–3 replicates; 6–9 animals/group). GO, gene ontology; BP, biological process; CC, cellular component; MF, molecular function; HP, Human Protein Atlas; REAC, Reactome.

To explore the relationship between changes in cocaine-induced chromatin accessibility and behavioral phenotypes, we focused first on the overlapping genes with cocaine-induced DARs in diestrus females and males (Figure 5A), since these groups showed stronger cocaine-induced conditioning responses in the CPP test (Figures 1C and 1D). In diestrus-male overlap, enrichment analysis revealed enrichment for genes related to GABAergic signaling, learning, and dendritic spines (Figure 5A). As examples, we observed changes in the promoter regions of three genes of interest: *Sp9*, encoding a transcription factor involved in MSN development^75^; *Fgfr1*, encoding fibroblast growth factor receptor 1, linked to addiction-related phenotypes^76,77^; and *GAD2*, encoding glutamate decarboxylase 2, the enzyme catalyzing GABA synthesis^78^ (Figure 5A and Table S4).

While there was substantial overlap in genes with cocaine DARs between diestrus females and males, the two groups may also reach a ‘‘susceptible state’’ through independent mechanisms. We therefore separately focused on genes with cocaine-induced DARs specific to each group. Male-specific genes were enriched for terms including positive regulation of gene expression, synaptic vesicle, and dopaminergic synapse (Figure 5B). While the dopaminergic synapse KEGG pathway was commonly enriched in all three groups (Figure S9B), a subset of dopaminergic-synapse-related genes undergo chromatin accessibility changes in males only, including the dopamine receptor-encoding genes *Drd1* (Figure 4D), *Drd2*, and *Drd3* (Figure 5B and Table S4). We also identified genes involved in GABAergic signaling with male-specific cocaine DARs, including *Gabra5*, encoding a GABA receptor whose activity in the human male NAc is associated with addiction^79^ (Figure 5B and Table S4).

Enrichment analysis of diestrus-specific genes revealed pathways including X-linked inheritance and estrogen-dependent gene expression (Figure 5C), indicating that both sex hormone signaling and the X chromosome are important features of the cocaine response in diestrus females. For example, cocaine increases chromatin accessibility in a region upstream of *Esr2*, encoding ERβ, specifically in diestrus females (Figure 5C and Table S4). We also observed diestrus-specific DARs at the promoters of *Ezh2*, encoding a histone methyltransferase, and *Egr2*, encoding early-growth response 2, an immediate-early gene critical for establishing cocaine-induced CPP^80^ (Figure 5C and Table S4). In summary, males and diestrus females exhibit similar changes in cocaine accessibility in genes related to GABAergic function but also have divergent responses, with male-specific DARs observed at dopamine receptor genes and diestrus-specific DARs observed at estrogen signaling-related genes.

To better understand why high-estrogenic females are less susceptible to cocaine’s conditioning effects (Figure 1D), we performed enrichment analysis of genes with proestrus-specific DARs (Figure 5D) and identified terms related to transcriptional and chromatin regulation (Figure 5D). We show three representative genes: *Ncoa1*, a transcriptional coregulator for steroid hormone receptors^81^; *Cacng2*, a calcium channel implicated in cocaine sensitization^82^; and *Npy2r*, a neuropeptide receptor whose polymorphisms in humans are associated with cocaine addiction^83^ (Figure 5D and Table S4). For all three genes, their putative promoter regions become less accessible in proestrus females after cocaine exposure (Figure 5D and Table S4). Notably, in controls, these regions are more accessible in proestrus compared to diestrus females and males, indicating that chromatin would typically gain accessibility to these regions as an element of a proestrus-specific chromatin state whereas, after cocaine exposure, these regions become more similar to diestrus females and males. Interestingly, when we overlapped proestrus cocaine-induced DARs that are less accessible after cocaine with DARs that are more accessible in proestrus vs. diestrus controls, we found enrichment for genes related to dendritic spines, synapse organization, and behavior (Figure S12). This interruption of proestrus DARs by cocaine exposure indicates a competition between cocaine-induced and proestrus-state-induced chromatin regulation, which may be protective for proestrus females by limiting the effects of cocaine on neuronal chromatin organization.

### Cocaine disproportionately leads to chromatin opening on the X chromosome in diestrus females

**Figure 6.**
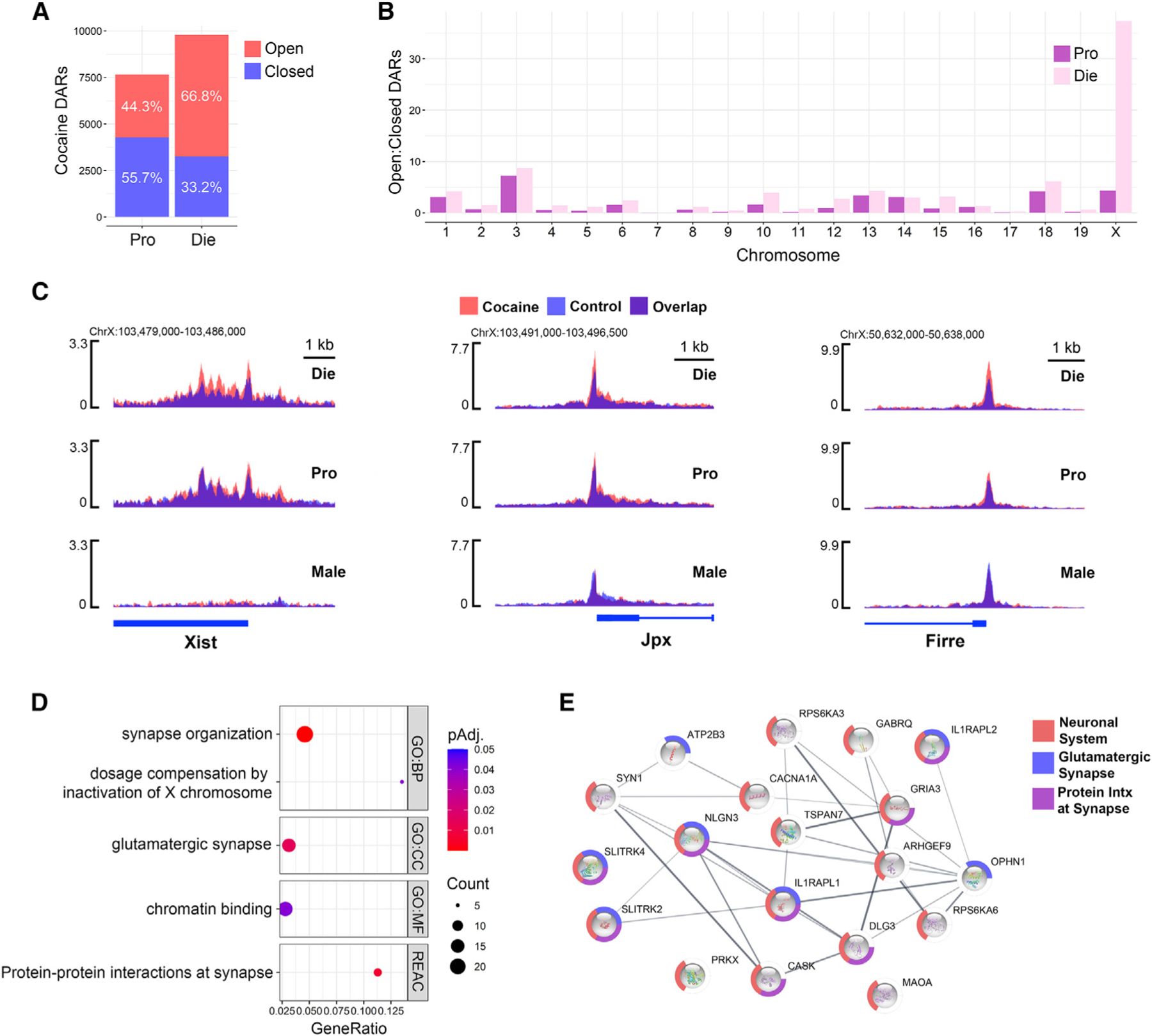
The role of the X chromosome in cocaine response is estrous cycle dependent (A) Among cocaine-induced differentially accessible regions (DARs), more chromatin regions become open (red) than closed (blue) in diestrus, while the opposite occurs in proestrus. (B) Across chromosomes, the ratio of open to closed cocaine-induced DARs is only skewed on the X chromosome for diestrus but not proestrus. (C) SparK plots of group-average normalized ATAC-seq reads (n = 2–3 replicates or 6–9 animals/group) are shown for the transcription start sites of the X-linked long non-coding RNAs Xist (left), Jpx (middle), and Firre (right). (D and E) For X-linked genes with diestrus cocaine-induced DARs: (D) enrichment analysis shows select gene ontology (GO) terms for biological process (BP), cellular component (CC), and molecular function (MF), together with Reactome (REAC) pathways. Colors indicate adjusted p values, and dot size corresponds to gene count. (E) Reactome pathways were visualized using String, with nodes corresponding to genes color coded by pathway and edges corresponding to protein-protein interactions between gene products.

Corroborating the notion that the proestrus phase is protected from the effects of cocaine on chromatin, we found that, in addition to having fewer cocaine-induced DARs than diestrus, proestrus-specific DARs are mostly regions that are less accessible after cocaine (55.7%). By contrast, the majority of diestrus-specific DARs are more accessible after cocaine (66.8%, Figure 6A). This pattern was most striking in gene promoters, where 83% of proestrus-specific DARs lose accessibility and 88% of diestrus-specific DARs gain accessibility after acute cocaine (Figure S13A). Moreover, gene promoters that become less accessible in proestrus after cocaine are enriched for terms and pathways related to neuronal function, addiction, and estrogen signaling (Figure S13B). To explore the preponderance of diestrus-specific DARs and given the enrichment for the X-linked inheritance term in diestrus-specific genes (Figure 5C), we assessed whether this ratio of open to closed DARs varied by chromosome. Indeed, we found that this ratio was particularly skewed on the X chromosome in diestrus females where practically all of the chromatin changes were characterized by more accessible chromatin, which was not the case in proestrus females (Figure 6B). Surprisingly, X-linked genes whose chromatin was impacted by cocaine in diestrus females included the same lncRNAs whose expression was disrupted by ELS in females (Figure 3E and Table S2), including Xist, Jpx, and Firre (Figure 6C and Table S4). In each case, cocaine increased chromatin accessibility at promoters in diestrus females only, and the corresponding *Xist* and *Ftx* gained-open regions contained the AP1 binding motif (Table S6). The same pattern of cocaine-induced, diestrus-specific promoter accessibility was also observed in autosomal genes involved in X inactivation, with expression altered by ELS in females (Figure S5A and Table S2), including *Ythdc1*, *Lrif1*, and *Smchd1* (Figure S14 and Table S4). In addition, we found that X-linked genes with cocaine-induced DARs in diestrus were enriched for synapse-related terms and pathways (Figure 6D). Reactome pathway analysis revealed enrichment of three neuronal pathways: neuronal system, glutamatergic synapse, and protein interactions at synapse, including *Syn1* and *Gria3* that are critical for synaptic plasticity (Figure 6E). In summary, cocaine exposure affects chromatin regulation of the X chromosome preferentially in diestrus females, affecting genes involved in dosage compensation and synaptic function, which can, in part, explain the susceptibility of diestrus females to the conditioning effects of cocaine.

### X chromosome inactivation: The mechanistic link between the effects of ELS and ovarian hormones on female-specific cocaine responses

**Figure 7.**
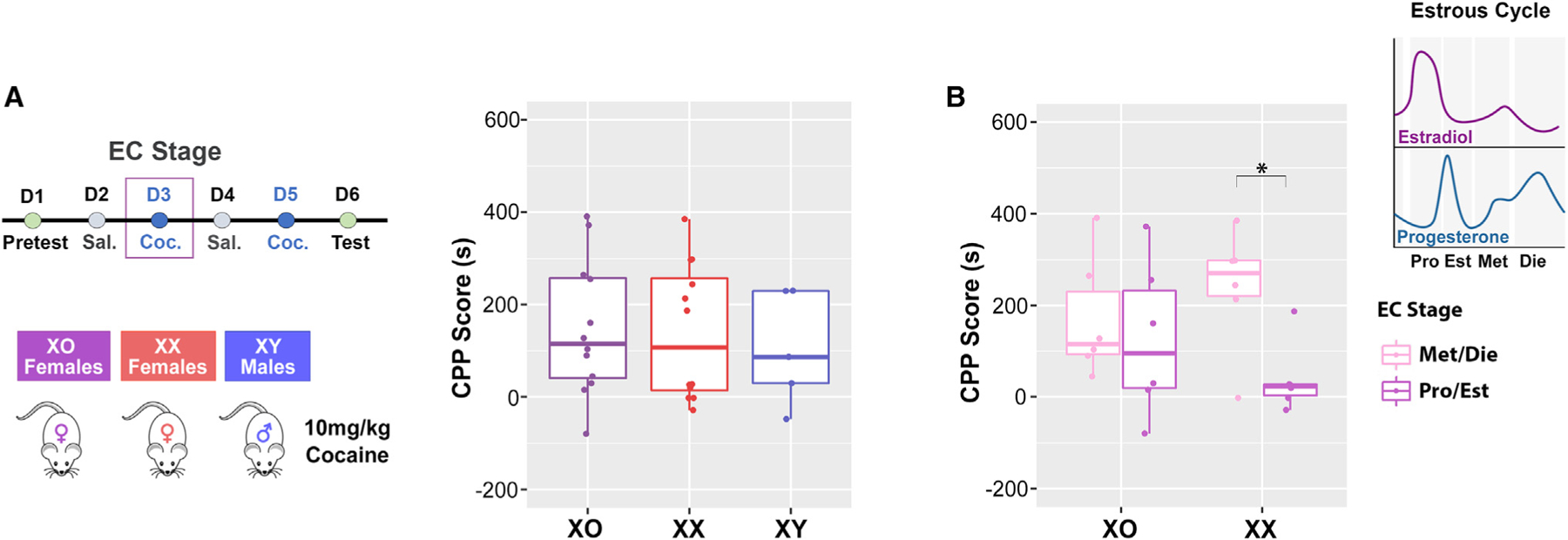
The estrous cycle’s effect on cocaine CPP requires Xi chromosome (A) The CPP test was performed with 39,XO females, XX females, and XY males (left) and analyzed in all three groups using one-way ANOVA (n = 12 females/group; n = 5 males/group, right). (B) The analysis was repeated in female mice with two-way ANOVA and group and estrous cycle (EC) stage as factors (n = 6 mice/stage/group). *p < 0.05, Welch’s post hoc two-sample t test. Box plots: box, 1st–3rd quartile; horizontal line, median; whiskers, 1.5× IQR. Pro, proestrus; Est, estrus; Met, metestrus; Die, diestrus.

So far, we identified two shared mechanisms, related to ERβ signaling and X chromosome inactivation, between the two susceptible female groups, control (low-estrogenic) diestrus females (Figures 5C, 6C, and 6D) and ELS-exposed females (Figure 3), revealing female-specific transcriptional mechanisms underlying cocaine-induced preference. ERβ signaling was previously implicated in cocaine-induced responses in female mice,^4^ but possible involvement of the female-unique phenomenon of X chromosome inactivation was particularly intriguing, as it was not previously explored. Thus, to directly assess the importance of Xi for cocaine’s effect in female mice, we performed another CPP experiment comparing genetically modified female mice with only one (active) X chromosome (39,X0), wild-type female mice (40,XX), and malemice (40,XY) ofthe same genetic background(Figure7A). Animals in each group, on average, developed a preference for the cocaine-paired compartment, and we did notdetecta difference in CPP scores between the three groups (Figure 7A). Within females, however, we found a significant effect of the estrous cycle (Figure 7B), with females in the low-estrogenic phase (Met/Die) on the first day of cocaine exposure exhibiting higher CPP scores than females in the high-estrogenic phase (Pro/Est), similar to what we showed previously in control C57BL/6J mice (Figures 1D and S1B). However, following up on this result with a post hoc test, we found that this effect could only be detected in the XX group while it was absent in the 39,XO group, indicating that the estrous cycle’s effect on cocaine CPP requires the presence of the second (Xi) X chromosome in females. Furthermore, these data provide the functional link between the effects of ELS and ovarian hormones on X-inactivation-relevant gene regulation and cocaine CPP phenotype.

## DISCUSSION

In this study, we show that two sex-specific risk factors for CUD, ELS and ovarian hormone status, induce sex-specific responses to cocaine in mice at both the behavioral and molecular level. Although cocaine can induce CPP in both sexes, the effect is more consistent in males, while it varies with the estrous cycle stage and is more strongly affected by ELS in females. We observe both shared and distinct effects of ELS and ovarian hormone status on transcriptional regulation in the NAc, and we link them to sex-specific responses to cocaine.

In control animals, we show that males consistently exhibit cocaine-induced conditioning with a 10 mg/kg dose, which was expected because this dose was based on previous research performed predominantly in males.^27^ The result in females, however, requires more careful consideration. Satta et al.,^4^ who used a 5 mg/kg dose of cocaine, also reported less consistent cocaine CPP effect in females. This varied response in females is consistent with reports that cocaine’s effects vary with the estrous cycle stage.^2,5^ However, the well-established finding is that estrogen facilitates cocaine-induced dopamine release and enhances cocaine self-administration during high-estrogenic phases (typically proestrus or estrus).^5,84^

Here, however, we report that experiencing a high-estrogenic state during the first cocaine exposure weakens the conditioning response in females. This response may reflect a protective effect of estrogen against cocaine-induced synaptic plasticity in the NAc, independent of the acute, dopamine-related reinforcing effect of estrogen. Low-estrogenic females, by contrast, seem to be the most susceptible to cocaine-induced CPP, as observed at 10 mg/kg dose as well as at the 2.5 mg/kg dose that is insufficient to induce CPP in males. One explanation for this is the negative affective state in mice during diestrus, in which we and others showed consistently higher anxiety indices compared to high-estrogenic, proestrus females.^19,24,85^ Consistent with this, earlier studies demonstrate that the first cocaine exposure has acute anxiolytic effects,^86^ which may facilitate association of cocaine with improved affective state in mice. This is further consistent with evidence in humans that women initiate drug use as a coping strategy to deal with anxiety and depression.^84^

Thus, cocaine’s effect on CPP is more complex in females than in males and we observe both protective and predisposing effects of ovarian hormones, depending on the estrous cycle phase. Interestingly, ELS erases the effect of the cycle and makes all females susceptible to cocaine CPP, along with males at their otherwise subthreshold dose. This effect of ELS partially mimics the effect of early stress seen in humans where both sexes show susceptibility to ELS enhancing addiction risk,^87^ but women are more strongly affected than men.^84^ Another finding requiring consideration is the preference in ELS males for lower doses over high doses of cocaine, which we found is consistent with an inverted U-shaped cocaine dose-response curve previously reported following adult stress exposure in male mice.^28^

We performed a gene expression study to address the underlying molecular mechanism for the sex-specific effect of ELS on cocaine-induced behavioral phenotypes. Two earlier studies characterized gene expression changes in the NAc after ELS,^20,21^ albeit using different stressors (limited bedding with or without maternal separation) and different developmental time points (P10–P17 or P2–P10). It is therefore unsurprising that our gene expression data differ as well. However, we do observe shared neurodevelopment-related genes and, importantly, all paradigms, and ours in particular, show a stronger effect of ELS on NAc gene expression in females than in males.^20,21^ In addition to the female-biased response, we also observe decreased sex differences in NAc gene expression following ELS, similar to the effect of adolescent social stress on the NAc transcriptome^56^ and mimicking the pattern found in our behavioral CPP data.

Two important findings help explain the female-biased effect of ELS on NAc gene expression and cocaine-induced CPP. First, we see enrichment of genes involved in estrogen signaling in response to ELS in females (but not in males), consistent with the role of NAc ERβ in cocaine-induced CPP development in female mice.^4^ Second, we find an overwhelming response of X chromosome-linked genes to ELS in females, including an intriguing increased expression of genes involved in X chromosome inactivation. Both of these mechanisms, acting separately or in concert, can induce a female-specific response to ELS of relevance to addiction. Specifically, downregulated genes in females are involved in synaptic function pointing toward altered regulation of synaptic plasticity after ELS. On the contrary, upregulated genes are involved in transcriptional and chromatin regulation, which are important elements of cocaine’s acute and chronic effects.

While ELS effectively nullifies the effect of ovarian hormone status on cocaine-induced CPP, these hormones have a critical effect on cocaine CPP in females under basal conditions. Thus, we chose to explore chromatin regulation in the NAc to address the molecular mechanisms through which ovarian hormones contribute to a female-specific cocaine response. While chromatin regulation in the NAc is strongly implicated in cocaine-induced adaptations underlying addiction-related behaviors, previous studies were almost exclusively performed in males.^17^ However, we previously reported sex- and estrous cycle-dependent chromatin regulation in the related brain area, the ventral hippocampus.^19^ Now, we demonstrate that acute cocaine treatment induces sex- and estrous cycle-dependent changes in neuronal chromatin within the NAc, providing a molecular substrate underlying sex-specific cocaine responses. Interestingly, we observe that cocaine, similar to ELS, reduces sex differences in NAc neuronal chromatin organization.

While many genes and pathways affected by cocaine are shared between males and females, the sex- and estrous cycle-specific molecular features of our ATAC-seq data reveal candidate sex-specific transcriptional mechanisms underlying cocaine-induced responses. First, we show enrichment of AP1 binding sites within NAc chromatin regions that gain accessibility in response to cocaine. While work in male animals demonstrated the role of Fos proteins in the cocaine response,^62,88–91^ our data provide experimental evidence of the genome-wide enrichment of Fos binding sites in chromatin opening following cocaine treatment in both sexes. In terms of sex specificity, AP1 sites were overwhelmingly enriched within promoter regions during diestrus, the low-estrogenic state that is most susceptible to developing cocaine preference. While the observed chromatin changes are induced by acute cocaine treatment, we also see indications that they are relevant to chronic cocaine exposure. We show that AP1 binding sites that gained chromatin accessibility in response to acute cocaine strongly overlapped with ΔFosB binding, a Fos protein extensively linked to chronic cocaine response.^62–67^ Considering that this overlap was most profound in diestrus females, we propose that the low-estrogenic state in females interacts with acute cocaine exposure and primes chromatin for later binding of ΔFosB and other molecular adaptations associated with chronic cocaine exposure. Accordingly, while cocaine-induced chromatin changes are associated with differential gene expression in the NAc, many cocaine-induced DARs do not correspond to immediate gene expression changes but may ‘‘prime’’ the genome for later transcriptional responses. In addition, a recent study showed that estrogen withdrawal increases ΔFosB expression in the NAc,^92^ consistent with our hypothesis that a physiological drop in estrogen (in diestrus) can ‘‘prime’’ chromatin for cocaine-induced long-term plasticity in the NAc.

Interestingly, we observed more overlaps in chromatin changes between diestrus females and males, the two groups ‘‘more susceptible’’ to cocaine CPP. These overlaps include addiction-related biological pathways such as dendritic spine density, learning, and GABAergic synapse. However, our data also indicated that these two groups may achieve a similar behavioral phenotype through different mechanisms. For instance, the enrichment of dopaminergic synapse-related genes has been frequently reported by previous studies, again largely performed in males.^93,94^ While our data confirm that the dopaminergic pathway is shared across sexes, we observed male-specific chromatin changes in genes encoding dopamine receptors, which are likely important for sex-specific cocaine-induced responses and could not have been anticipated by earlier male-focused studies. Interestingly, diestrus-specific cocaine-induced DARs are enriched in X-linked and estrogen-signaling-related genes, including diestrus-specific chromatin opening within the *Esr2* gene encoding ERβ. These two mechanisms, related to ERβ signaling and X chromosome-linked gene expression, are shared between the two susceptible female groups, control diestrus and ELS-exposed females, revealing female-specific transcriptional mechanisms underlying cocaine-induced preference.

We would specifically like to emphasize a coincidence of increased expression and increased chromatin accessibility of the X-linked lncRNAs (Xist, Jpx, Firre) in females after ELS and after cocaine exposure in diestrus, respectively. Considering the critical roles of these lncRNAs in dosage compensation,^36–38,40^ our findings indicate a disruption in Xi regulation in the two cocaine-susceptible female groups. In general, X chromosome dysregulation can contribute to altered neuronal function underlying the ELS-induced and diestrus-driven susceptibility to cocaine, given the over-representation of genes related to brain development and function on the X chromosome.^95^ Accordingly, the role of the X chromosome in cocaine response was previously addressed by the four-core genotypes model, showing that the XX genotype, regardless of sex hormones, affects cocaine vulnerability.^96^ Here, however, we used 39,XO mice to reveal the functional role of Xi in response to cocaine within naturally cycling female mice. Unlike humans with Turner syndrome (45,X0) whose ovaries are either missing or not functioning properly, 39,XO mice have functional ovaries and undergo the estrous cycle.^97^ Using the 39,XO model, we thus show that having only one X chromosome nullifies the protective effect of physiologically high estrogen levels in female mice. Moreover, similar to the effect of ELS or physiological estrogen withdrawal, lack of Xi leads to enhanced susceptibility to cocaine-induced conditioning response in females.

In addition to female-specific susceptibility, we would also like to address the molecular mechanisms that may underlie the ‘‘protective effect’’ of estrogen against cocaine-induced synaptic plasticity in the NAc. Interestingly, acute cocaine exposure leads to preferential closing of chromatin in proestrus, the opposite of what we observed in the susceptible diestrus group. This interruption of proestrus-specific open chromatin regions by cocaine exposure indicates a competition between cocaine- and estrogen-induced chromatin regulation, which may be protective for proestrus females by limiting the effects of cocaine on neuronal chromatin organization. However, we would like to reiterate that previous studies showed estrogen-induced enhancement of cocaine’s reinforcing effects.^84^ Considering that this effect may be mediated via fast estrogen signaling,^2^ it is not necessarily inconsistent with estrogen’s protective effect on cocaine-induced chromatin plasticity, which is slower and likely to have more long-term consequences.

Substance use disorders involve multiple components from motivational aspects and initiation of drug use to adaptive changes in the brain that underlie chronic drug use and addiction. Our study highlights that cocaine use in women and other menstruating individuals may involve complex effects of ovarian hormones that interact with internal factors (such as negative affective state) and external risk factors such as stress and, importantly, we reveal the molecular substrates through which female-specific factors can induce stronger and longer-lasting sex-specific responses to cocaine. By revealing functional link(s) between female-specific phenomena such as regulation of X chromosome inactivation and cocaine conditioning responses, our findings offer a molecular framework to enable understanding of sex-specific mechanisms underlying CUD, facilitating the development of sex- and gender-informed treatments for CUS.

### Limitations of the study

While we provide molecular substrates through which ELS and ovarian hormones interact to induce female-specific cocaine responses, including evidence for the role of X chromosome inactivation in this process, this study also opens many new questions that will need to be addressed by future studies. First, we show that ELS ‘‘erases’’ the effect of the estrous cycle on cocaine CPP, but the mechanism underlying this effect is unknown. ELS may affect estrous cycling in female mice and change ovarian hormone levels or, alternatively, brain responsiveness to ovarian hormones may be changed in response to ELS. A separate, carefully designed study is needed to address this question. Additionally, future work should address the upstream mechanism(s) mediating the effects of ELS on gene expression, and this may involve manipulation of transcription factors whose binding sites are enriched near stress-induced DEGs. While we observed enrichment of chromatin-related factors in stress-induced DEGs, ATAC-seq or other epigenomics assays following the MSUS paradigm will further reveal the mechanism(s) underlying MSUS-induced gene expression changes. Further studies would also benefit from single-cell approaches that can pinpoint specific cellular populations involved in sex-specific cocaine responses. The ΔFosB ‘‘priming’’ hypothesis in females is very intriguing, and further studies utilizing alternative addiction-related paradigms including cocaine self-administration and chronic cocaine treatments will further reveal the role of the female-specific and estrogen-dependent transcriptional program(s) involved in chronic cocaine use and addiction. Many additional data analyses can be performed, and we believe that the rich data produced in this article will help move the field forward, particularly regarding the less-understood female-specific mechanisms involved in cocaine reward.

## STAR★METHODS

### RESOURCE AVAILABILITY

#### Lead contact

Further information and requests for resources and reagents should be directed to and will be fulfilled by the lead contact, Marija Kundakovic (mkundakovic@fordham.edu).

#### Materials availability

This study did not generate new unique reagents.

#### Data and code availability

RNA-seq and ATAC-seq data are available from the Gene Expression Omnibus and are publicly available as of the date of publication. Accession numbers are listed in the key resources table. This paper does not report original code. Any additional information required to reanalyze the data reported in this paper is available from the lead contact upon request.

### EXPERIMENTAL MODEL AND STUDY PARTICIPANT DETAILS

#### Animals

For ELS experiments, five-week-old male (n = 18) and four-week-old female (n = 36) C57BL/6J mice were obtained from Jackson Laboratories. After two weeks of habituation period, mating pairs (n = 18) were formed consisting of one male and two female mice and were separately housed. After mating, n = 34 of the subsequent litters were used in this study. Litters were counted at P0, and litters were randomly assigned to either Con or MSUS groups (n = 17 litters/group). Litters were weaned at P28 and housed in cages with mice of the same sex and group (n = 5 per cage). For acute cocaine treatment (ATAC-seq) experiments, four-week-old male (n = 18) and female (n = 48) C57BL/6J mice were obtained from Jackson Laboratories. After habituation, female animals underwent estrous cycle monitoring for approximately two weeks (see *Estrous Cycle*), prior to acute cocaine treatment at P52–54. For the X chromosome-focused CPP experiment, 39,XO females (n = 12), XX females (n = 12), and XY males (n = 5) of the same genetic background (C57Bl/6J x CBA/CaGnLeJ, Strain #036414) were obtained from Jackson Laboratories. The genotype of animals was confirmed in-house using the established qPCR assay (Jackson Laboratories Protocol #35972) performed on DNA isolated from tail clippings. All animals used in the study were housed in same-sex cages (n = 3–5/cage), were kept on a 12h:12h light:dark cycle, were given *ad libitum* access to food and water, and were allowed to habituate to the facility for at least two weeks prior to experiments. All animal procedures were approved by the Institutional Animal Care and Use Committee at Fordham University.

### METHOD DETAILS

#### Early-life stress (ELS)

To administer ELS, a well-established maternal separation combined with unpredictable maternal stress (MSUS) protocol was used.^23–25^ Litters chosen for ELS (MSUS group) were separated from the dams for 3 h each day from P1-P14. Control litters (Con group) did not undergo any manipulations during this time. The separation window was changed each day to ensure that the stress was unpredictable. During separation, the dam was placed in a temporary cage with food and water. Dams were also subjected to unpredictable stress, either restraint stress (20min) or forced swim stress (2min) at a random time during the 3-h separation window, in order to prevent the occurrence of compensatory maternal care following separation, which was reported to occur.^108^ We confirmed that our unpredictable stress protocol disrupts maternal behavior for at least 1 h after the mother is returned to the home cage (Figure 1B).

#### Maternal observations

Maternal behavior was scored from Day 1 through Day 6 postpartum in both Con and MSUS dams following the separation of MSUS dams from their pups. Every 3 min during the 1-h observation period, the maternal behavior during the time of inspection was recorded for each cage. Cataloged behaviors included nursing, arched-back nursing, licking and grooming, in contact with pups (not nursing), out of nest, eating, drinking, and nest building.^24,26,109^

#### Cocaine-induced conditioned place preference

From P54-P60, Con (n = 50 females, 30 males) and MSUS (n = 30 females, 30 males) animals underwent cocaine-induced conditioned place preference (CPP) testing. At this point mice were further grouped by cage into a low dose (LD) group, receiving a subthreshold dose of 2.5 mg/kg i.p. cocaine, and high dose (HD) group, receiving a standard dose of 10 mg/kg i.p. cocaine on conditioning days (Days 3 and 5, Figure 1C). For the X chromosome experiment, females (n = 12/group) and males (n = 5) underwent the same CPP test with only the standard 10 mg/kg dose of cocaine. Animals in the X chromosome experiment were 11–13 weeks old at the time of testing, and the smaller number of males were included as a reference group to provide a comparison of the CPP phenotype to the C57Bl/6J cohort of animals across sex. Our CPP apparatus is custom made (Maze Engineers; Skokie, IL) and consists of two conditioning chambers separated by a corridor with sliding walls. The conditioning chambers were designed to contain both visual and tactile cues, with one chamber containing striped walls and metal bars on the floor (Compartment 1), and the other containing gray walls and a plastic-holed floor (Compartment 2, Figure S15A). A pilot experiment with a separate cohort of animals was used to confirm that neither conditioning chamber was generally preferable to the mice in the absence of pairing stimuli (Figure S15B). The testing paradigm involved a pretest day, followed by 4 conditioning days (alternating between saline and cocaine), and finishing with a test day (Figure 1). During both pretest and test days, the walls were removed so that the mice could freely roam either chamber in the apparatus. During the conditioning days, mice were administered either cocaine or saline and the walls were inserted into the apparatus to confine the mice to one compartment. We used an unbiased CPP protocol in which, following injection of the mice with cocaine (2.5 or 10 mg/kg, i.p.), half were confined to Compartment 1 and the other half were confined in Compartment 2. Cocaine preference was established by calculating the CPP score^110^ which was the time the animal spent in the cocaine-paired compartment on test day minus the time spent in the cocaine-paired compartment on pretest day. This score accounts for any baseline compartment preference or aversion in individual animals.

#### Estrous cycle staging

Female mice were assigned to four possible stages of the cycle (proestrus, estrus, metestrus, and diestrus) based on cellular composition in vaginal smears^19^ (Figure S1A). Vaginal lavage smears were taken by gently aspirating distilled water into the vaginal opening with a sterile pipette and applying the smear onto a microscope slide. After drying, slides were stained with 0.1% crystal violet solution and visualized under a light microscope. The proestrus phase is marked by predominantly nucleated epithelial cells, while estrus is characterized by mostly cornified epithelial cells. Metestrus and diestrus phases are characterized by cornified and nucleated epithelial cells, as well as leukocytes, with diestrus smears containing a far greater number of leukocytes than metestrus. For animals undergoing CPP testing, the vaginal smears were obtained each day during the 6-day period of the CPP test, following the pretest, conditioning, or test session. For these experiments, the statistical analysis of behavioral data was based on the classification of females into two groups: Pro/Est animals (occupying proestrus, estrus, or transitionary phases, Figure S1B), and Met/Die animals (occupying metestrus, diestrus, or transitionary phases, Figure S1B). For these classifications, females were grouped based on their estrous cycle stage on the first day of cocaine exposure due to the tendency of female rodents to experience disruption of the estrous cycle following cocaine administration.^111^ While for behavioral cohorts we needed to combine the estrous cycle stages to increase power of analysis (Figure 1D), for the ATAC-seq experiments we were able to track the estrous cycle more comprehensively and include more specific groups, with better defined ovarian hormone levels: proestrus (high-estrogen, low-progesterone) and diestrus (low-estrogen, high-progesterone) females, which mimic human follicular and luteal phases, respectively.^19^ This more comprehensive procedure included daily vaginal smears for approximately two weeks (or three cycles) to establish a predictive cycling pattern for each animal. This allowed us to predict the estrous cycle stage of female animals and ensure an adequate number of proestrus and diestrus females on the day of cocaine administration. Postmortem vaginal smears were taken from these animals to confirm their estrous cycle stage. Finally, for the X chromosome-focused CPP experiment, the estrous cycle was tracked comprehensively for two weeks to ensure that all animals were cycling normally prior to behavioral testing; however, the groups were still merged into Pro/Est and Met/Die for the analysis of CPP data, as was done for the ELS CPP experiment, in order to increase power of the analysis.

#### Quantification of serum estradiol

Serum levels of estradiol from a cohort of 11-week old female C57Bl6/J mice (n = 16) were quantified using a BioVision mouse ELISA kit (K3830) (Figure S1B). Trunk blood was collected at the time of sacrificing into 1.5-mL tubes and was allowed to clot at room temperature for 2 h. Serum was separated by centrifugation (1503 × *g*, 20 min, 4°C) and stored at −80°C prior to the ELISA procedure. ELISA was performed using the manufacturer’s instructions, and samples were measured alongside the included standards in duplicate. The absorbance was measured using a SpectraMax plate reader and serum concentrations of estrogen were determined using the generated standard curve.

#### Drugs

Cocaine-HCl was purchased from Fagron (800006) under a Controlled Substance Schedule II license granted by the United States Drug Enforcement Agency (RK0524238) and New York State Department of Health (04C0098).

#### Acute cocaine administration

To determine the sex-specific and estrous cycle-dependent effects of acute cocaine exposure on neuronal chromatin accessibility, groups consisting of proestrus females, diestrus females, and males aged P52–54 (aligning with the age of CPP-tested mice) were given an acute dose of 10 mg/kg i.p. cocaine (100 μL of solution containing 2.5 mg/mL of Cocaine-HCl dissolved in ddH_2_O) and sacrificed by cervical dislocation 1 h later, while controls received no cocaine and served as the 0-h baseline (n = 6–9/group/time-point). Whole brains were removed, bilateral NAc were dissected immediately and snap frozen in liquid nitrogen.

#### RNA-seq

At P50, just prior to the onset of CPP behavioral testing, a subset of male and female MSUS and Con mice (n = 6/group/sex) were sacrificed by cervical dislocation, brains were removed and snap frozen in hexane. The estrous cycle of female animals was determined via vaginal smear cytology, and an equal proportion of high (n = 3/group; proestrus, estrus, or transitionary phases) and low (n = 3/group; metestrus, diestrus, or transitionary phases) estrogenic females were included, to account for the effect of the estrous cycle on gene expression.^19^ Bilateral NAc were dissected inside a cryostat using a brain matrix and 1.5 mm biopsy tissue punch. RNA was isolated from the NAc using the RNeasy Micro Kit (Qiagen). RNA quality was assessed using the Fragment Analyzer (Agilent) and all RNA samples had an RQN of ≥9.0. RNA samples were quantified using the Qubit RNA High-Sensitivity assay (Thermo Fisher Scientific). cDNA libraries were prepared from RNA samples using the RNA HyperPrep Kit with RiboErase (KAPA Biosystems). First, rRNA was depleted from the RNA samples by hybridizing complementary DNA oligonucleotides to rRNA followed by treatment with RNase H and DNase. The efficiency of rRNA depletion was confirmed using qRT-PCR targeting the 28S rRNA transcript before and after RiboErase treatment (Primer sequences: Forward – 5′-CCCATATCCGCAGCAGGTC-3′, Reverse – 5′-CCAGCCCTTAG AGCCAATCC-3′). Following rRNA depletion, RNA was fragmented at 94°C for 5 min in the presence of Mg^2+^ and first and second cDNA strands were synthesized, followed by A-tailing. Single-indexed adaptors (KAPA) were ligated and libraries were amplified by PCR. After bead-based purification, library quality was determined with the Fragment Analyzer (Agilent), and quantification was performed using the Qubit High-Sensitivity DNA assay (Thermo Fisher Scientific). 100 bp, paired-end sequencing was performed on the Nova-Seq 6000 instrument at the New York Genome Center.

#### RNA-seq analysis

Sequences obtained from the RNA-seq experiment were adapter trimmed and aligned using Star^98^ to the mouse reference genome (mm10 including small contigs) using the GENCODE M15 gtf file. Following alignment, differential gene expression analysis was performed using DESeq2,^99^ and significant DEGs were considered as P_adj_ < 0.10 using DESeq2’s default correction for multiple testing (Benjamini-Hochberg). Differential expression between MSUS and Con groups was determined for males and females separately. For the female analysis, estrous cycle status of females was incorporated into the model. A Group*Sex interaction analysis was also performed in DEseq2 to detect genes for which the change in expression between MSUS and Con groups was unequal between males and females. Gene coexpression clustering analysis across the four experimental conditions was performed using Clust,^30^ which allows the identification of genes with similar changes in expression patterns across groups. Notably, while this analysis does not require genes to be DEGs in order to be included in a cluster, the cluster genes we identified were typically also DEGs in the female analysis but not in the male analysis (Table S2F), as expected, while the benefit of Clust is illustrating these gene expression differences in females in the context of sex differences. Motif analysis on genes identified in Clust clusters was performed using Homer.^105^ Gene list enrichment analyses were performed using gProfiler.^100^ Enrichment analysis was also performed using GSEA^34^ with a ranked gene list and results were plotted using EnrichmentMap,^112^ AutoAnnotate, and GeneMANIA apps^113^ in Cytoscape.^101^ By utilizing a ranked list of all detected genes, GSEA analysis also allowed us to incorporate male gene expression data and continue focusing on sex differences and expression patterns across the experimental conditions. Aligned RNA-seq read data was plotted using SparK.^102^ Read numbers and quality control metrics for RNA-seq libraries are shown in Table S7.

#### Fluorescence-activated nuclear sorting (FANS)

For the ATAC-seq experiments, the purification of neuronal nuclei from bulk frozen brain tissue was carried out using a protocol we previously established.^114^ Dissected bilateral NAc tissue was pooled from 3 animals of the same group (forming 2–3 biological replicates per group, see Acute Cocaine Administration) and frozen in liquid nitrogen. Tissue was later homogenized in a tissue douncer. Nuclei were extracted by ultracentrifugaton through a gradient of sucrose for 1 h at 24,400rpm in a 4°C Beckman ultracentrifuge. Following centrifugation through the sucrose gradient, the nuclei-containing pellet was resuspended in DPBS and incubated with a monoclonal mouse antibody targeting NeuN, a nuclear protein expressed in neurons, conjugated with the Alexa Fluor 488 fluorophore (1:1000; Millipore, MAB 377X). Nuclei were also incubated with DAPI (1:1000; ThermoFisher Scientific, 62248) prior to sorting and then filtered through a 35-μm cell strainer. Nuclei samples were sorted using the FACSAria instrument (BD Sciences) at the Albert Einstein College of Medicine Flow Cytometry Core Facility. In order to determine the gating strategy (Figure S16), the following three controls were used: 1. An isotype control incubated with IgG1-AlexaFluor-488 and DAPI, 2. A DAPI-only control, and 3. A NeuN-AlexaFluor-488-only control. Gates were adjusted based on events collected from control samples in order to select single nuclei from cellular debris and clumped nuclei, and to separate the NeuN+ (neuronal) and NeuN− (non-neuronal) population. From each sample we collected 68,000–75,000 NeuN+ nuclei in BSA-coated tubes containing 200μL DPBS.

#### ATAC-seq

Following FANS, neuronal nuclei from the NAc were centrifuged at 2900 x g for 10 min at 4°C. Then, to perform transposition of chromatin for ATAC-seq,^19,53,115^ the supernatant was removed after centrifugation and the nuclei pellet was resuspended in 50μL of a transposase reaction mix which contained 2.5μL Tn5 Transposase enzyme and 25μL of 2xTD reaction buffer (Nextera DNA Library Preparation Kit). Transposition occurred at 37°C for 30 min, after which the DNA was purified from the samples using the MinElute PCR Purification Kit (Qiagen). A PCR reaction with the following components was used to index and amplify the transposed DNA: 10μL DNA, 5μL PCR Primer Cocktail (Illumina), 25μL NEBNext High-Fidelity 2x PCR Master Mix (New England Biolabs), and 5μL of Nextera i5 and i7 indexed amplification primers (Illumina). The cycling conditions were as follows: 72°C for 5 min, 98°C for 30 s, then 5 cycles of 98°C for 10 s, 63°C for 30 s, and 72°C for 1 min. After 5 PCR cycles, a 20-cycle qPCR reaction was performed with 5μL of PCR-amplified libraries to determine the optimal number of PCR cycles needed to achieve appropriate library concentration without losing library complexity (typically 4–5 additional cycles). Following PCR, libraries were purified with the MinElute PCR Purification kit (Qiagen). Library quality was determined with the Bioanalyzer High-Sensitivity DNA assay (Agilent), and quantification was performed with the Qubit High-Sensitivity DNA assay (Life Technologies) and by qPCR (KAPA Biosystems). 100bp, paired-end sequencing was performed on the Nova-Seq 6000 instrument at the New York Genome Center.

#### ATAC-seq analysis

Following sequencing, adapters were trimmed and sequences were aligned to the mouse reference genome (mm10 including small contigs) using BWA-MEM.^103^ Peak-calling was then performed with MACS2, as reported previously.^53^ After peak calling, peaks belonging to the Y chromosome, mitochondrial DNA, or blacklisted regions, as well as peaks not shared by at least two replicates were removed from the analysis. The featureCounts function of the Rsubreads package^107^ was used to count the reads in each peak across replicates. The resulting count matrix was analyzed using DESeq2^99^ to determine differential chromatin accessibility between cocaine and control groups separately for males, diestrus females, and proestrus females. Significant peaks were annotated to the nearest gene using ChIPpeakAnno.^104^ Motif analysis on cocaine DARs was performed using Homer.^105^ Gene enrichment analyses were performed using gProfiler^100^ and Reactome enrichment was performed in Cytoscape.^101^ Track plots of ATAC-seq data were generated using SparK.^102^ Heatmaps and histograms of ATAC-seq reads within cFos- and ΔFosB-bound regions were generated using ngs.plot.^106^ Statistical significance for overlapping genomic regions was evaluated by MCFDR,^116^ performed using the Galaxy HypERβrowser.^117^ Read numbers and quality control metrics for ATAC-seq libraries are shown in Table S8.

### QUANTIFICATION AND STATISTICAL ANALYSIS

For the ELS CPP experiment, we performed a three-way analysis of variance (ANOVA) with group, sex, and dose as factors. To analyze the effect of the estrous cycle within control females, we performed a two-way ANOVA with dose and estrous cycle stage as factors. For the X chromosome-focused CPP experiment, we first performed a one-way ANOVA based on genotype; then, within females, we performed a two-way ANOVA with genotype and estrous cycle as factors. For all multivariate ANOVA tests, we first evaluated whether the interaction term was significant, and if it was not significant we dropped the interaction term from the model, followed by an analysis of the main effects of the factors. For the within-female XO CPP analysis, we performed post-hoc t-tests to understand the genotype by estrous cycle interaction effect. For maternal behavior scores, we performed repeated-measures ANOVA tests with day and group as factors, and we also analyzed the average maternal behavior scores across the 6 days using t-tests. Statistical assessment of gene overlaps was performed using Fisher’s Exact Test (FET). The results of these tests were considered significant at a p < 0.05. Boxplots depict the 1st–3rd quartile, with the horizontal line denoting the median and the whiskers denoting 1.5 times the inter-quartile range. Statistical details can be found in the relevant results section and in the figure legends. The analyses were performed in R version 4.2.2 and the graphs were generated using the R ggplot2 package.

## Supplementary Material

Table S1

Table S3

Figures S1-S16

Table S5

Table S6

Table S7

Table S8

Table S4

Table S2

## Figures and Tables

**Table:** KEY RESOURCES TABLE

REAGENT or RESOURCE	SOURCE	IDENTIFIER
Antibodies		

Mouse anti-NeuN-AlexaFluor-488	Millipore	Cat#MAB377X; RRID: AB_2149209
Mouse IgG1-k-AlexaFluor-488	Millipore	Cat#FCMAB310A4; RRID: AB_10806480

Chemicals, peptides, and recombinant proteins		

Cocaine-HCl	Fagron	Cat#800006

Critical commercial assays		

RNA HyperPrep Kit with RiboErase	KAPA Biosystems	Cat#KK8560
Nextera DNA Library Preparation Kit	Illumina	Cat#FC-121-1030

Deposited data		

ATAC-seq and RNA-seq data	This paper	GEO: GSE229696
cFos ChIP-seq data	Malik et al. (^68^)	GEO: GSE60192
ΔFosB CUT&RUN data	Yeh et al. (^54^)	GEO: GSE197668
GRCm38 mm10 mouse reference genome	Genome Reference Consortium	ncbi.nlm.nih.gov/assembly/GCF_000001635.20/
GENCODE M15 gtf file	Genome Reference Consortium	https://www.gencodegenes.org/mouse/release_M15.html

Experimental models: Organisms/strains		

Mouse: C57BL/6J	The Jackson Laboratory	Strain#000664; RRID: IMSR_JAX:000664
Mouse: C57Bl/6J x CBA/CaGnLeJ	The Jackson Laboratory	Strain#036414; RRID: IMSR_JAX:036414

Software and algorithms		

Star	Dobin et al. (^98^)	https://github.com/alexdobin/STAR
DEseq2	Love et al. (^99^)	https://bioconductor.org/packages/release/bioc/html/DESeq2.html
Clust	Abu-Jamous et al. (^30^)	https://github.com/BaselAbujamous/clust
gProfiler	Raudvere et al. (^100^)	https://biit.cs.ut.ee/gprofiler/gost
GSEA	Subramanian et al. (^34^)	https://www.gsea-msigdb.org/gsea/index.jsp
Cytoscape	Shannon et al. (^101^)	https://cytoscape.org/
SparK	Kurtenbach et al. (^102^)	https://github.com/harbourlab/SparK
BWA-MEM	Li (^103^)	https://github.com/lh3/bwa
ChIPpeakAnno	Zhu et al. (^104^)	https://bioconductor.org/packages/release/bioc/html/ChIPpeakAnno.html
Homer	Heinz et al. (^105^)	http://homer.ucsd.edu/homer/motif/
ngs.plot	Shen et al. (^106^)	https://github.com/shenlab-sinai/ngsplot
Rsubreads	Liao et al. (^107^)	https://bioconductor.org/packages/release/bioc/html/Rsubread.html

## Highlights

- Early-life stress more strongly alters gene expression and cocaine reward in females
- Ovarian hormone status affects cocaine-induced chromatin and behavioral changes
- Acute cocaine causes sex- and estrous cycle-dependent increased accessibility of ΔFosB sites
- X chromosome inactivation mechanistically links female-specific risk factors and cocaine use
