# Supplementary material for: Early-life stress and ovarian hormones alter transcriptional regulation in the nucleus accumbens resulting in sex-specific responses to cocaine
Source: Cell Rep. Author manuscript; Available in PMC 2023 Dec 28. (PMC10753961; doi:10.1016/j.celrep.2023.113187)
Supplement: Figures S1-S16 [file NIHMS1936794-supplement-Figures_S1-S16.pdf]

Cell Reports, Volume *42*

Supplemental information

Early-life stress and ovarian hormones alter transcriptional regulation in the nucleus accumbens resulting in sex-specific responses to cocaine

Devin Rocks, Ivana Jaric, Fabio Bellia, Heining Cham, John M. Greally, Masako Suzuki, and Marija Kundakovic

Supplementary Information

This PDF file includes:

Supplementary Figures

Supplementary Figure 1, related to Figure 1: Estrous cycle stage determination and corresponding estradiol levels

Supplementary Figure 2, related to Figure 2: Sex differences in NAc gene expression

Supplementary Figure 3, related to Figure 2: Early-life stress and sex interact to alter NAc gene expression

Supplementary Figure 4, related to Figure 2: Shared effects of early-life stress on female NAc gene expression across study paradigms

Supplementary Figure 5, related to Figure 2: Examples of genes in the identified coexpression clusters

Supplementary Figure 6, related to Figure 2: Yy1 and Klf-family transcription factors are downregulated in the female NAc by early-life stress.

Supplementary Figure 7, related to Figure 3: Early life stress alters genes involved in X-inactivation and X-linked genes involved in synaptic function in the female NAc

Supplementary Figure 8, related to Figure 4: Acute cocaine exposure reduces sex differences in NAc chromatin accessibility

Supplementary Figure 9, related to Figure 4: Group overlaps and shared enrichment of genes with cocaine differentially accessible regions (DARs)

Supplementary Figure 10, related to Figure 4: Association of chromatin accessibility changes with gene expression in the NAc

Supplementary Figure 11, related to Figure 4: Accessible chromatin after acute cocaine in the NAc overlaps more strongly with ΔFosB-bound regions than cFos-bound regions

Supplementary Figure 12, related to Figure 5: Regions more accessible in proestrus female controls become less accessible after acute cocaine exposure

Supplementary Figure 13, related to Figure 6: Chromatin regions surrounding gene promoters are less accessible after acute cocaine in proestrus females

Supplementary Figure 14, related to Figure 6: Promoter regions of autosomal genes involved in X-inactivation are more accessible after cocaine in diestrus females

Supplementary Figure 15, related to Figure 1: Cocaine conditioned place preference (CPP) paradigm

Supplementary Figure 16, related to Figure 4: Purification of neuronal (NeuN+) nuclei with fluorescence-activated nuclei sorting (FANS)

Supplementary Tables

Supplementary Table 1, related to Figure 1: Summary statistics for the cocaine CPP test (uploaded as a separate excel file)

Supplementary Table 2, related to Figure 2: Results of gene expression analyses in the NAc (uploaded as a separate excel file)

Supplementary Table 3, related to Figure 3: Gene set enrichment analysis (GSEA) Enrichment Map data for Females, Males, and Group x Sex interaction genes (uploaded as a separate excel file)

Supplementary Table 4, related to Figure 4: Results for differential chromatin accessibility analysis in the NAc (uploaded as a separate excel file)

Supplementary Table 5, related to Figure 4: Linear regression statistics for the association between chromatin accessibility and gene expression (uploaded as a separate excel file)

Supplementary Table 6, related to Figure 4: Cocaine differentially accessible regions (DARs) with AP-1 binding sites and DARs overlapping ΔFosB-bound regions (uploaded as a separate excel file)

Supplementary Table 7, related to Figures 2-3: RNA-seq data basic information for the early-life stress experiment (uploaded as a separate excel file)

Supplementary Table 8, related to Figures 4-6: ATAC-seq data basic information for the acute cocaine treatment experiment (uploaded as a separate excel file)

Supplementary Figure 1

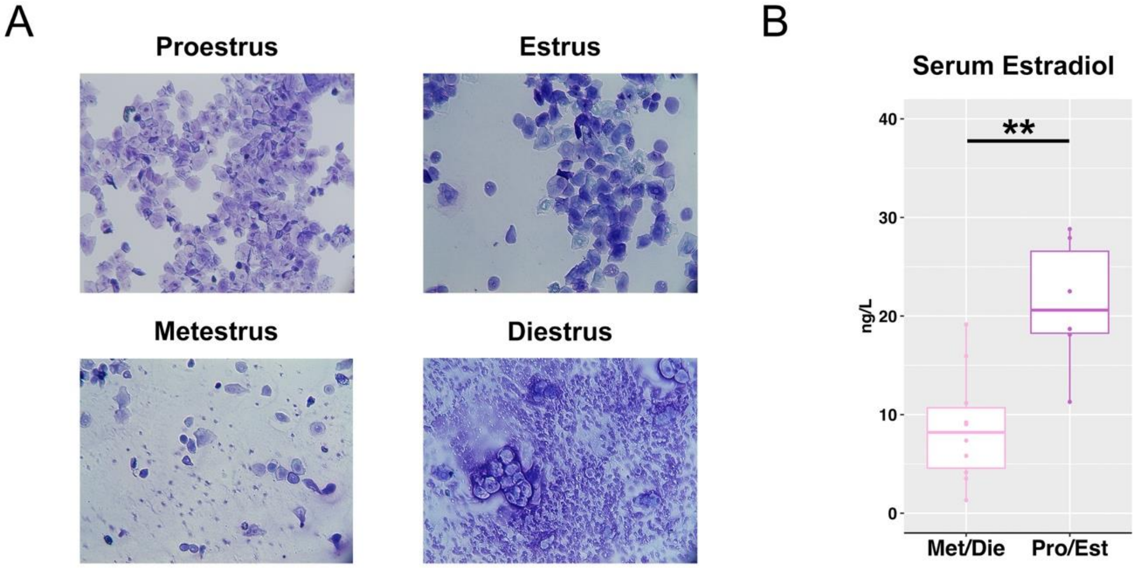

Supplementary Figure 1. Estrous cycle stage determination and corresponding estradiol levels. (**A**) Representative images of vaginal smears stained with crystal violet exhibiting the cell type composition characteristic of each of the four phases of the estrous cycle, including the proestrus phase characterized by mostly nucleated epithelial cells (top-left), the estrus phase characterized by mostly cornified epithelial cells (top-right), and the metestrus (bottom-left) and diestrus phases (bottom-right) which have both nucleated and cornified epithelial cells as well as leukocytes, with diestrus being distinct in having a greater proportion of leukocytes. (**B**) ELISA analysis demonstrates animals grouped into the Proestrus/Estrus group (Pro/Est) have higher serum estradiol levels than those in the Metestrus/Diestrus group (Met/Die). ** $p < 0.01$; Welch two-sample T-test. Box plots (box, 1st–3rd quartile; horizontal line, median; whiskers, 1.5× IQR).

Supplementary Figure 2

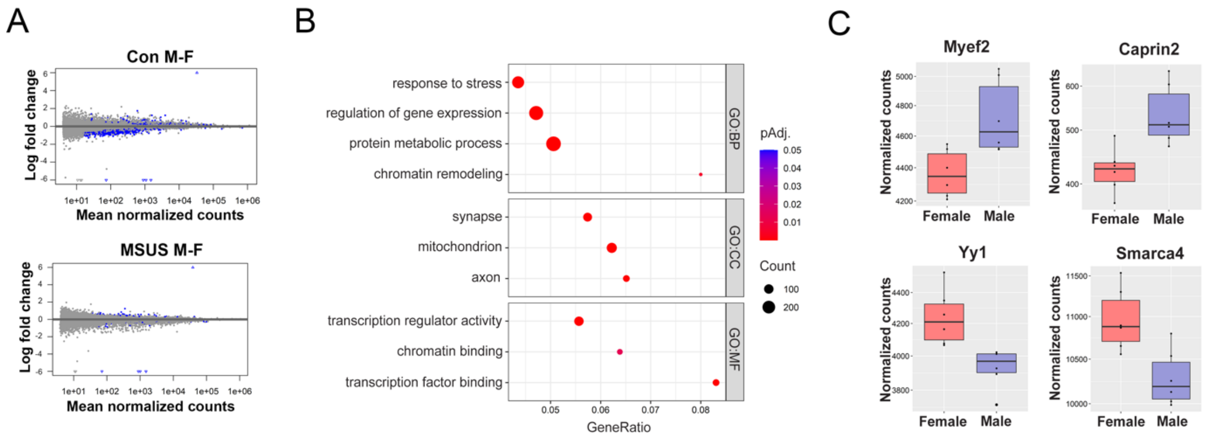

Supplementary Figure 2. Sex differences in NAc gene expression. (**A**) RNA-seq was performed on the NAc from female (F) and male (M) mice of the control (Con) and maternal separation combined with unpredictable maternal stress (MSUS) group (n = 6 animals/sex/group). Volcano plots depict differentially expressed genes across sex in the Con group (n = 1508), and in the MSUS group (n = 163); blue dots represent significant genes (p_{adj} < 0.1). (**B**) Dotplot depicting select gene ontology (GO) terms for biological process (BP), cellular compartment (CC), and molecular function (MF) significantly enriched in the genes (n=1508) differentially expressed between Con males and females in the NAc. Colors indicate adjusted *p*-values and dot size corresponds to gene count. (**C**) Example genes with differential expression across sex in the Con NAc including *Myef2* (p_{adj} = 0.0978, top left) and *Caprin2* (p_{adj} = 0.0208, top right), more highly expressed in males than females, as well as *Yy1* (p_{adj} = 0.0663, bottom left) and *Smarca4* (p_{adj} = 0.0859, bottom right), more highly expressed in females than in males. Notably, the Yy1 transcription factor is implicated in driving the transcriptional effects of early-life stress in females (**Figure 2**). Box plots (box, 1st–3rd quartile; horizontal line, median; whiskers, 1.5× IQR). Normalized counts, count data normalized using DESeq2's median ratio normalization method.

Supplementary Figure 3

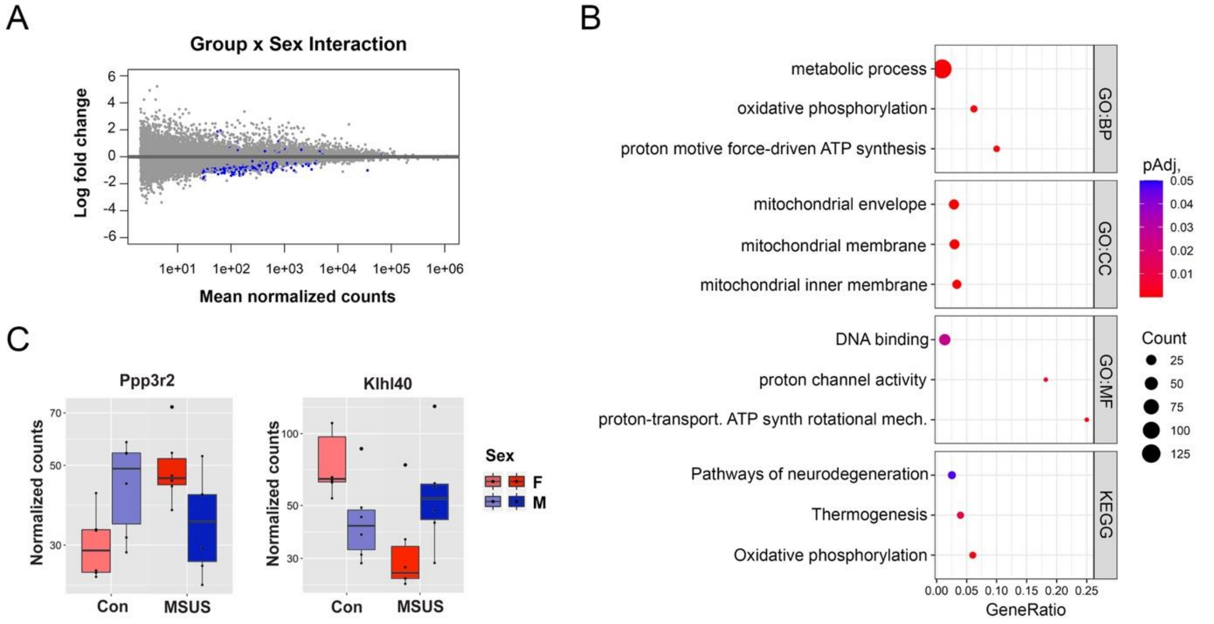

Supplementary Figure 3. Early-life stress and sex interact to alter NAc gene expression. (A) Volcano plot depicts differentially expressed genes identified in the Group x Sex interaction analysis (n = 455); blue dots represent significant genes ($p_{adj} < 0.1$). (**B**) Dotplot depicting select gene ontology (GO) terms for biological process (BP), cellular compartment (CC), and molecular function (MF), as well as KEGG pathways, significantly enriched in genes with a significant group by sex interaction. Colors indicate adjusted *p*-values and dot size corresponds to gene count. (**C**) Example genes include *Ppp3r2* ($p_{adj} = 0.0892$, left), which, on average, shows higher expression in females but lower expression in males after early-life stress, and *Klhl40* ($p_{adj} = 0.0898$, right), which, on average, shows lower expression in females but higher expression in males after early-life stress. Box plots (box, 1st–3rd quartile; horizontal line, median; whiskers, 1.5× IQR). Con, control group; MSUS, maternal separation combined with unpredictable maternal stress group; Normalized counts, count data normalized using DESeq2's median ratio normalization method.

Supplementary Figure 4

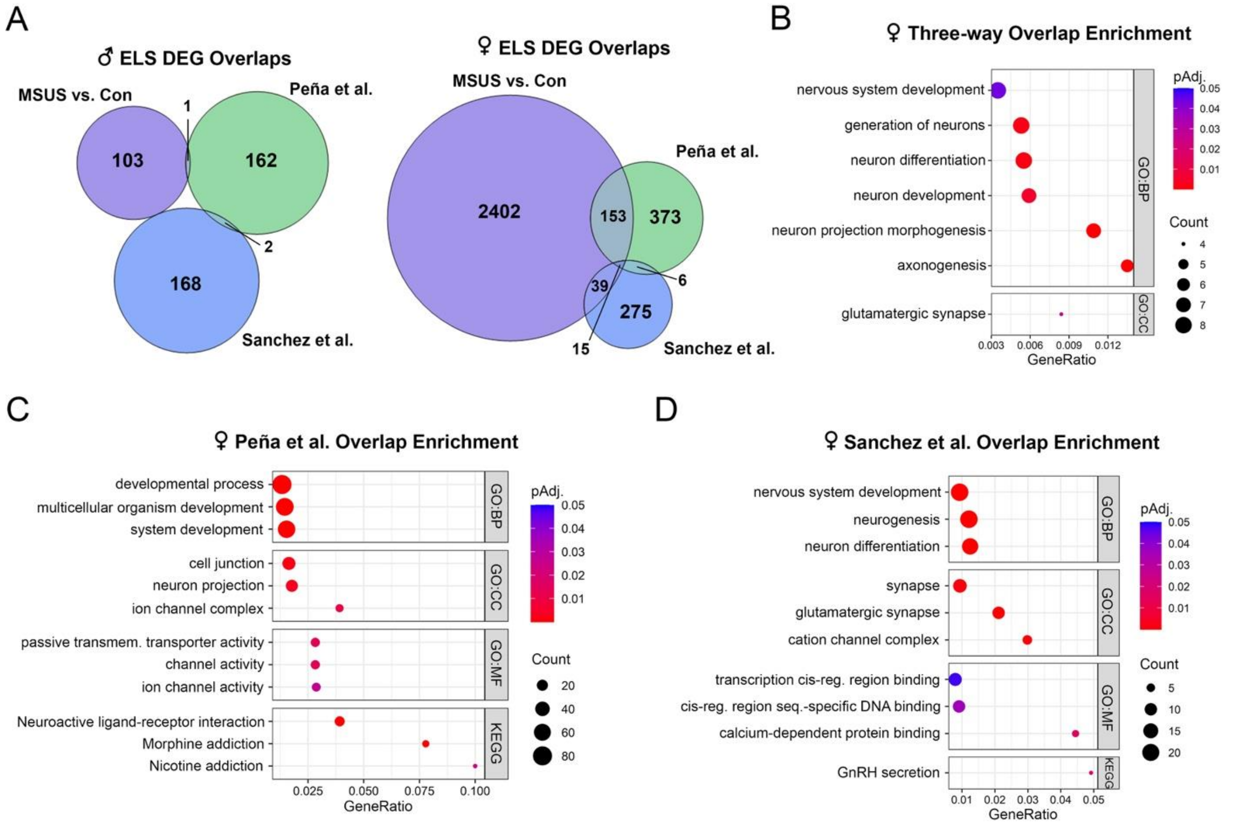

Supplementary Figure 4. Shared effects of early-life stress on female NAc gene expression across study paradigms. (**A**) Venn diagrams showing the overlap of differentially expressed genes (DEGs) altered by early-life stress in the NAc in this study (MSUS vs. Con), a study utilizing limited-bedding and maternal separation from postnatal day (P) 10-17 (Peña et al.[20]), and a study utilizing limited nesting and bedding from P2-P10 (Sanchez et al.[21]) in males (left) and females (right). A looser criterion for defining a DEG ($p_{nominal} < 0.05$), which was used by the other two studies, was applied to our data only in this instance to facilitate overlaps. (**B**) Dotplot depicting select gene ontology (GO) terms for biological process (BP) and cellular compartment (CC) significantly enriched in female DEGs shared between the three studies. (**C**) Dotplot depicting select GO terms for BP, CC, and molecular function (MF), as well as KEGG pathways, significantly enriched in female DEGs shared between this study and the Peña et al. study. (**D**) Dotplot depicting select GO terms for BP, CC, and MF, as well as KEGG pathways, significantly enriched in female DEGs shared between this study and the Sanchez et al. study. Dotplot colors indicate adjusted *p*-values and dot size corresponds to gene count. Con, control group; MSUS, maternal separation combined with unpredictable maternal stress group.

Supplementary Figure 5

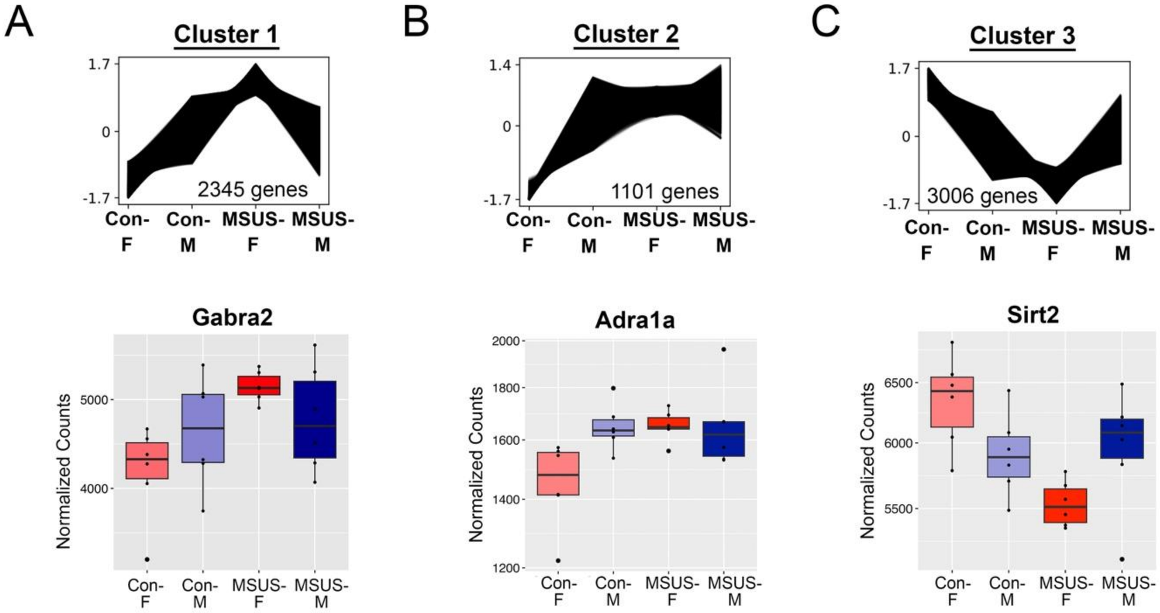

Supplementary Figure 5. Examples of genes in the identified coexpression clusters. For each gene coexpression clusters shown in **Figure 2** (top row) an example gene is shown with a box plot of normalized gene counts across the groups with the expected expression pattern (bottom row). (**A**) For example, *Cluster 1* includes *Gabra2*, encoding GABA-A receptor subunit 2, which is significantly more highly expressed in MSUS females compared to control females (p_{adj} = 0.000738, **Suppl. Table 2**). (**B**) In *Cluster 2*, *Adra1a*, encoding adrenergic receptor alpha 1a, is also significantly more highly expressed in MSUS females compared to control females (p_{adj} = 0.0341, **Suppl. Table 2**). (**C**) Finally, in *Cluster 3*, *Sirt2*, encoding sirtuin 2, a histone deacetylase, is significantly downregulated in MSUS females compared to control females (p_{adj} = 0.000313, **Suppl. Table 2**). Box plots (box, 1st–3rd quartile; horizontal line, median; whiskers, 1.5× IQR). Con-F, control female; Con-M, control male; MSUS-F, maternal separation combined with unpredictable maternal stress female; MSUS-M, maternal separation combined with unpredictable maternal stress male; Normalized counts, count data normalized using DESeq2's median ratio normalization method.

Supplementary Figure 6

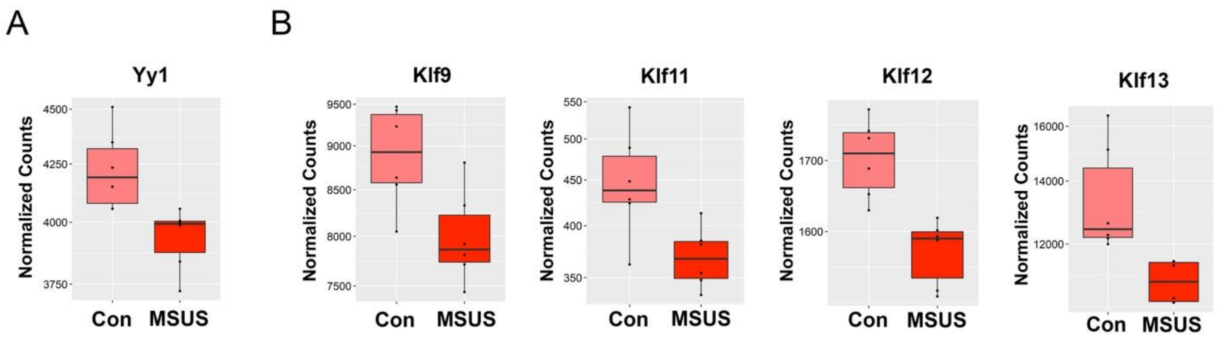

Supplementary Figure 6. Yy1 and Klf-family transcription factors are downregulated in the female NAc by early-life stress. (**A**) Normalized count plot of the gene encoding the Yy1 transcription factor (p_{adj} = 0.0360), whose binding sites are enriched in promoters of cluster 1 genes (**Figure 2**) are shown for the female group. (**B**) Normalized count plots of genes encoding Klf-family transcription factors, including *Klf9* (p_{adj} = 0.0498), *Klf11* (p_{adj} = 0.0116), *Klf12* (p_{adj} = 0.0266), and *Klf13* (p_{adj} = 0.00831), whose binding sites are enriched in promoters of cluster 3 genes (**Figure 2**) are shown for the female group. Box plots (box, 1st–3rd quartile; horizontal line, median; whiskers, 1.5× IQR). Con, control group; MSUS, maternal separation combined with unpredictable maternal stress group; Normalized counts, count data normalized using DESeq2's median ratio normalization method.

Supplementary Figure 7

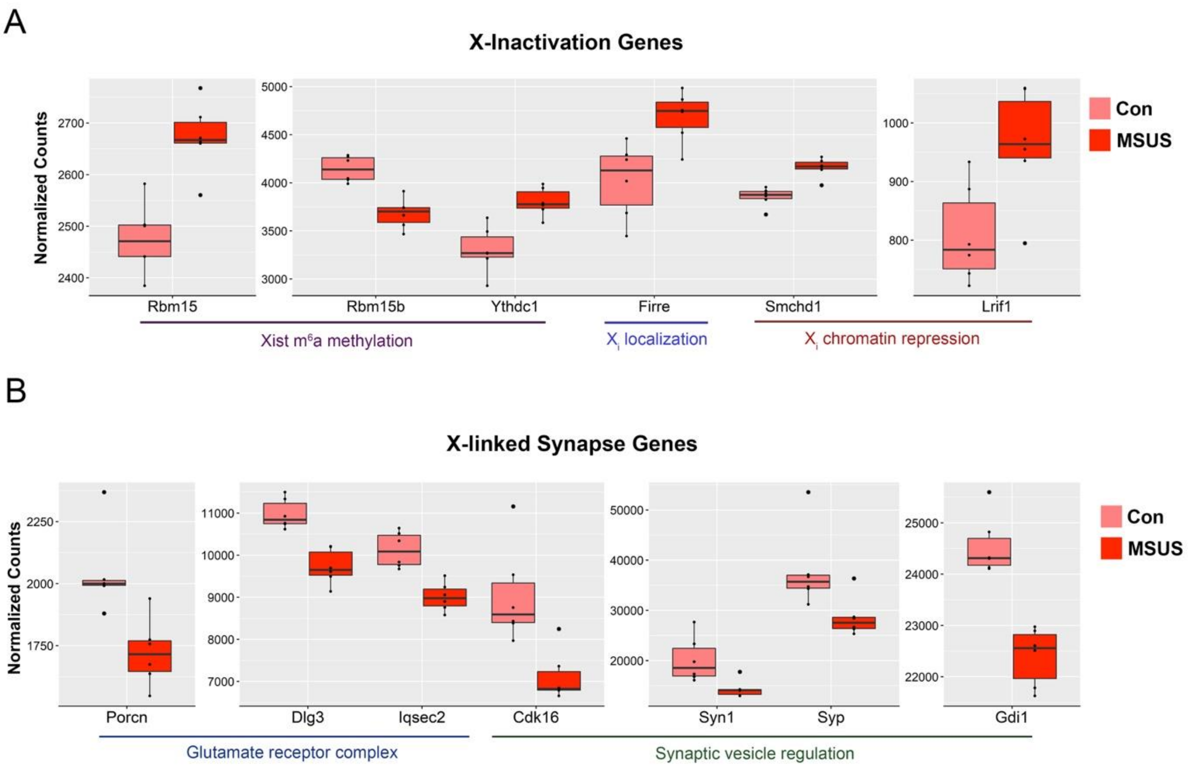

Supplementary Figure 7. Early-life stress alters genes involved in X-inactivation and X-linked genes involved in synaptic function in the female NAc. (**A**) Normalized count plots showing the altered expression of autosomal and X-linked genes involved in X-inactivation, including Xist m^{6}a methylation (*Rbm15*, p_{adj} = 0.0129; *Rbm15b*, p_{adj} = 0.000649; and *Ythdc1*, p_{adj} = 0.00524; autosomal), localization of the inactive-X chromosome (X_i) (*Firre*, p_{adj} = 0.0202); X-linked), and X_i chromatin repression (*Smchd1*, p_{adj} = 0.00693 and *Lrif1*, p_{adj} = 0.0239; autosomal; left to right) (**B**) Normalized count plots showing the altered expression of X-linked genes involved in synapse function, including genes encoding members of the glutamate receptor complex (*Porcn*, p_{adj} = 0.00769; *Dlg3*, p_{adj} = 0.000213; and *Iqsec2*, p_{adj} = 0.000164) and genes involved in synaptic vesicle regulation (*Cdk16*, p_{adj} = 0.00244; *Syn1*, p_{adj} = 0.00496; *Syp*, p_{adj} = 0.0178; and *Gdi1*, p_{adj} = 0.000180; left to right). Box plots (box, 1st–3rd quartile; horizontal line, median; whiskers, 1.5× IQR). Con, control group; MSUS, maternal separation combined with unpredictable maternal stress group; Normalized counts, count data normalized using DESeq2's median ratio normalization method.

Supplementary Figure 8

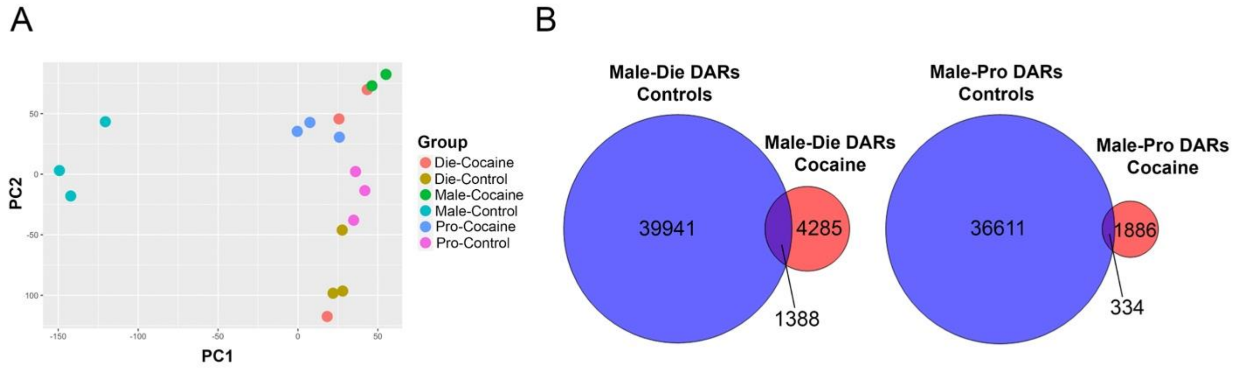

Supplementary Figure 8. Acute cocaine exposure reduces sex differences in NAc chromatin accessibility. (**A**) A PCA plot showing the clustering of ATAC-seq replicates across groups and treatment. While male and female controls are separated along the first principal component (PC1), cocaine-treated males and females cluster together more closely. (**B**) Venn diagrams depicting the overlap of differentially accessible chromatin regions (DARs) between males and diestrus females in the control and cocaine conditions (left) and between males and proestrus females in the control and cocaine conditions (right), illustrating that cocaine treatment produces a smaller, largely distinct set of sex DARs compared to those present in controls. Die, diestrus; Pro, proestrus; Male, males.

Supplementary Figure 9

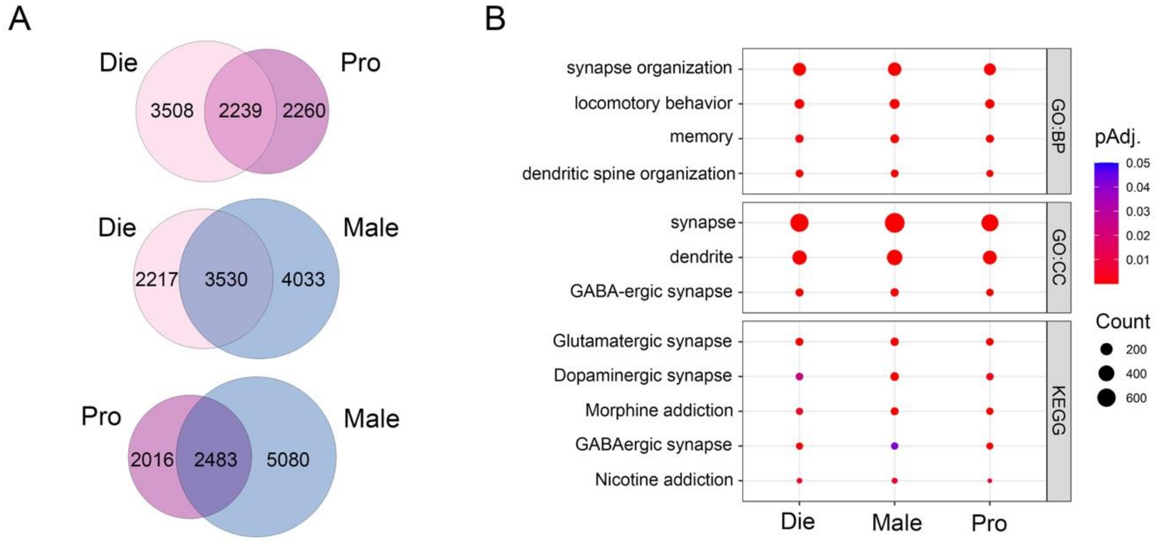

Supplementary Figure 9. Group overlaps and shared enrichment of genes with cocaine differentially accessible regions (DARs). (**A**) Overlap of genes annotated to cocaine-induced DARs in each group comparison. (**B**) For each group, dot plots show select gene ontology (GO) terms for biological process (BP) and cellular component (CC), as well as KEGG pathways enriched in genes annotated to cocaine-induced DARs. Colors indicate adjusted p-values and dot size corresponds to gene count. Die (light pink), diestrus; Pro (purple), proestrus; Male (blue), males.

Supplementary Figure 10

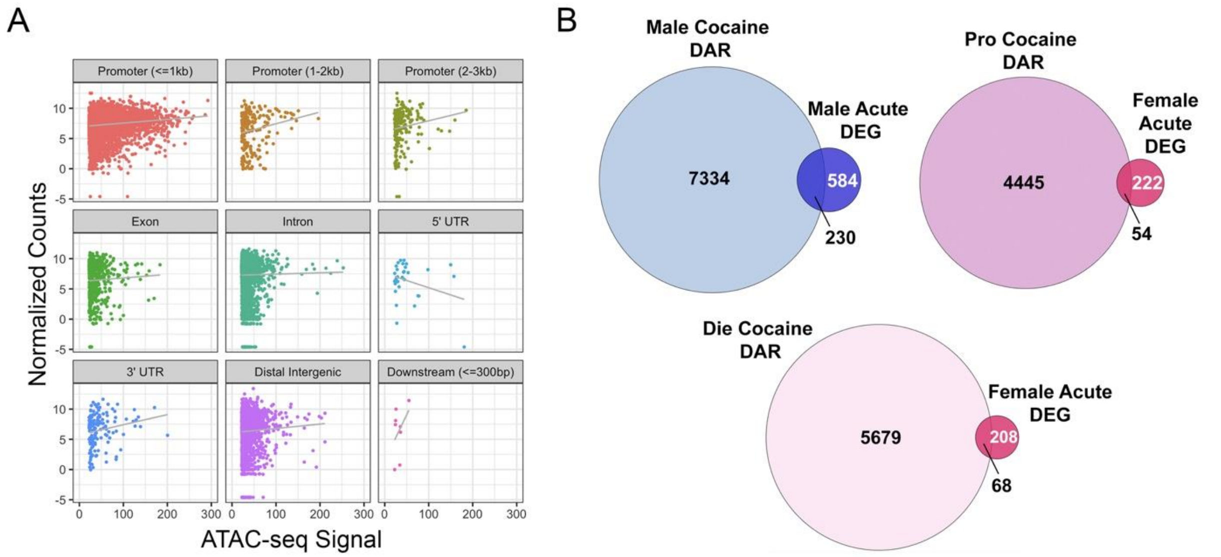

Supplementary Figure 10. Association of chromatin accessibility changes with gene expression in the NAc. (**A**) Linear regression of ATAC-seq signal in annotated peaks with RNA-seq counts demonstrate an association between ATAC-seq signal at promoter regions and gene expression levels. This plot shows a representative analysis in diestrus females; see **Suppl. Table 5** for detailed statistics of the linear regressions performed in each group. (**B**) Overlaps between genes with cocaine differentially accessible regions (DARs) in this study with differentially expressed genes (DEGs) identified after acute (1-hour) 7.5 mg/kg cocaine treatment in males and females by the Walker et al. study[56]. Note that in all cases female ATAC-seq data are segregated by the estrous cycle phase (proestrus or diestrus) while RNA-seq are derived from one (mixed stages) female group.

Supplementary Figure 11

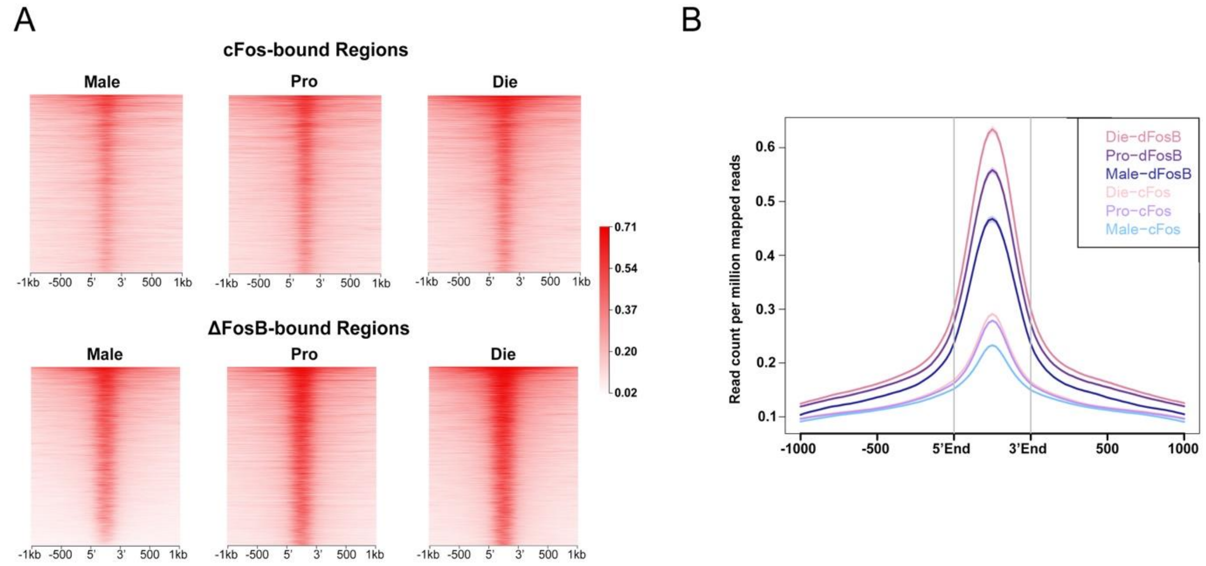

Supplementary Figure 11. Accessible chromatin after acute cocaine in the NAc overlaps more strongly with ΔFosB-bound regions than cFos-bound regions. (**A**) Heatmaps show the density of ATAC-seq reads in acute cocaine treated males (left) proestrus females (middle), and diestrus females (right) within regions that are bound by cFos (top) or ΔFosB (bottom). (**B**) A histogram representation of the data shown in (**A**), demonstrating a higher ATAC-seq signal in ΔFosB-bound regions than cFos-bound regions for all groups, especially diestrus females. Die, diestrus; Pro, proestrus; Male, males.

Supplementary Figure 12

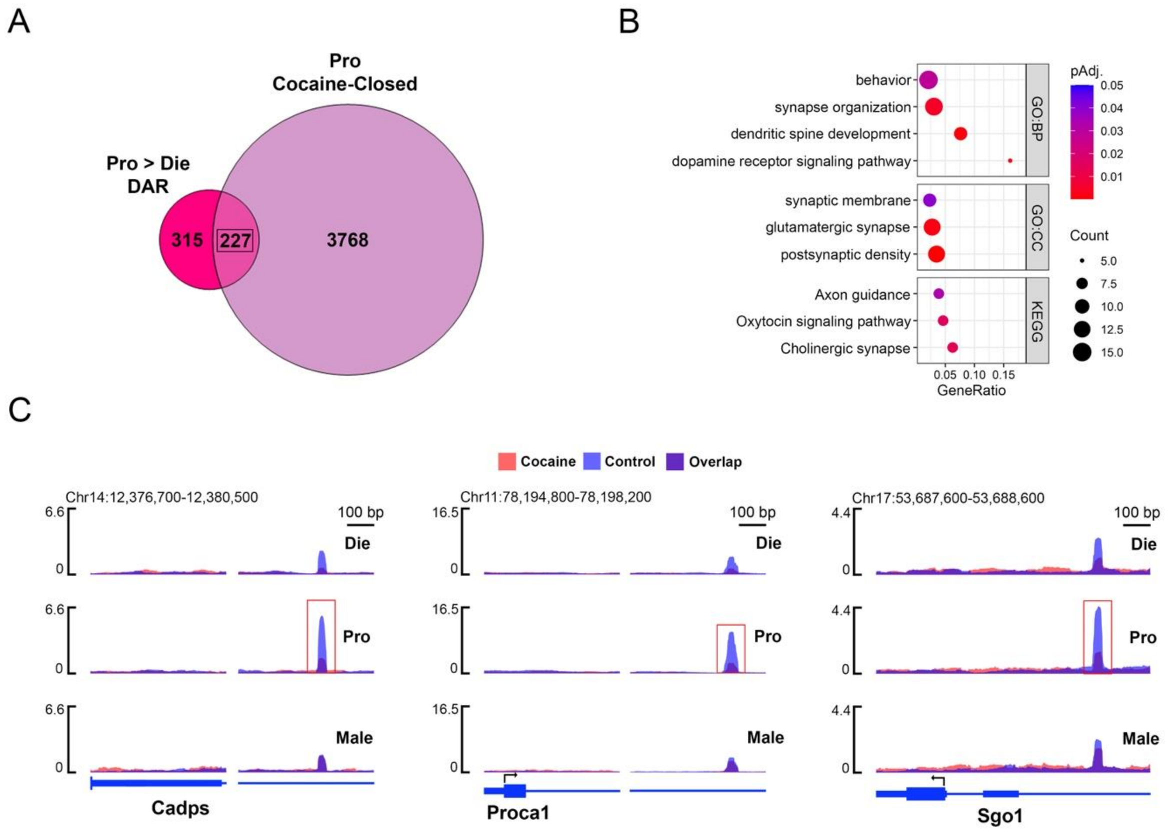

Supplementary Figure 12. Regions more accessible in proestrus female controls become less accessible after acute cocaine exposure. (**A**) A Venn diagram showing the overlap between differentially accessible regions (DARs) that are more accessible in proestrus than diestrus controls, with regions that are less accessible in proestrus females following acute cocaine exposure. (**B**) A dotplot depicting select gene ontology (GO) terms for biological process (BP) and cellular component (CC), as well as KEGG pathways, significantly enriched in the genes annotated to overlapping regions highlighted in (**A**). Dotplot colors indicate adjusted *p*-values and dot size corresponds to gene count. (**C**) SparK plots of group-average normalized ATAC-seq reads (n = 2-3 replicates or 6-9 animals/group) are shown for example regions from the overlap shown in (**A**), including regions near the transcription start sites (TSSs) of *Cadps* (left), *Proca1* (middle), and *Sgo1* (right), all of which are more accessible in proestrus controls before cocaine but lose accessibility in this group after cocaine. Die, diestrus; Pro, proestrus; Male, males.

Supplementary Figure 13

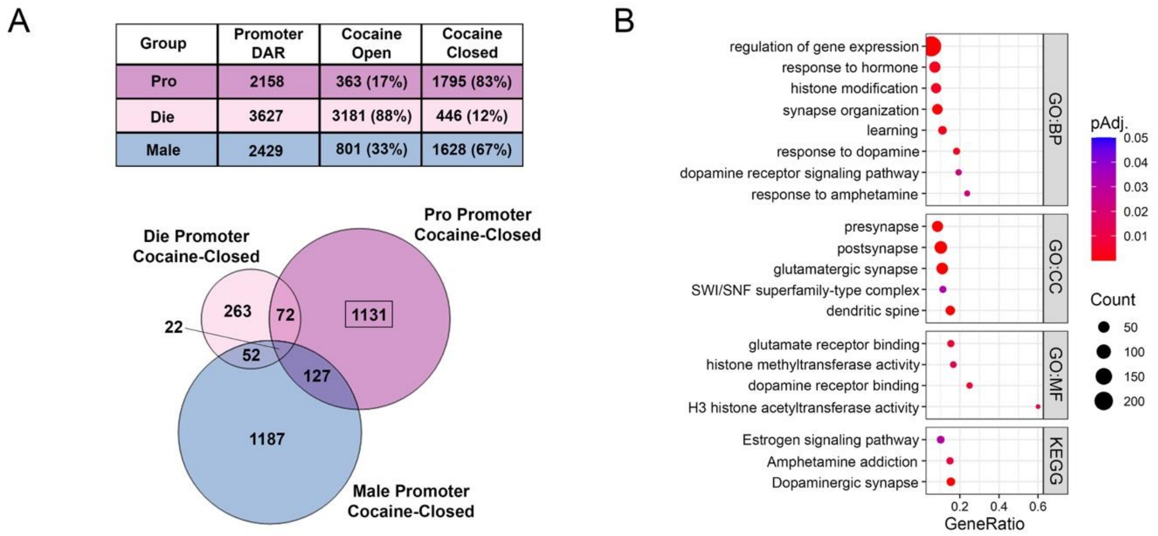

Supplementary Figure 13. Chromatin surrounding gene promoters are less accessible after acute cocaine in proestrus females. (**A**) A table showing the number of cocaine differentially accessible regions (DARs) that overlap gene promoters and the number of these regions that become more (Cocaine Open) or less (Cocaine Closed) accessible after cocaine in each group (top), as well as a Venn diagram showing the overlap of promoter DARs that are less accessible after cocaine in all three groups (bottom). (**B**) A dotplot depicting select gene ontology (GO) terms for biological process (BP), cellular component (CC), and molecular function (MF) as well as KEGG pathways, significantly enriched in the genes annotated to proestrus-specific promoter DARs that are less accessible after cocaine, highlighted in the Venn diagram shown in (**A**). Dotplot colors indicate adjusted *p*-values and dot size corresponds to gene count.

Supplementary Figure 14

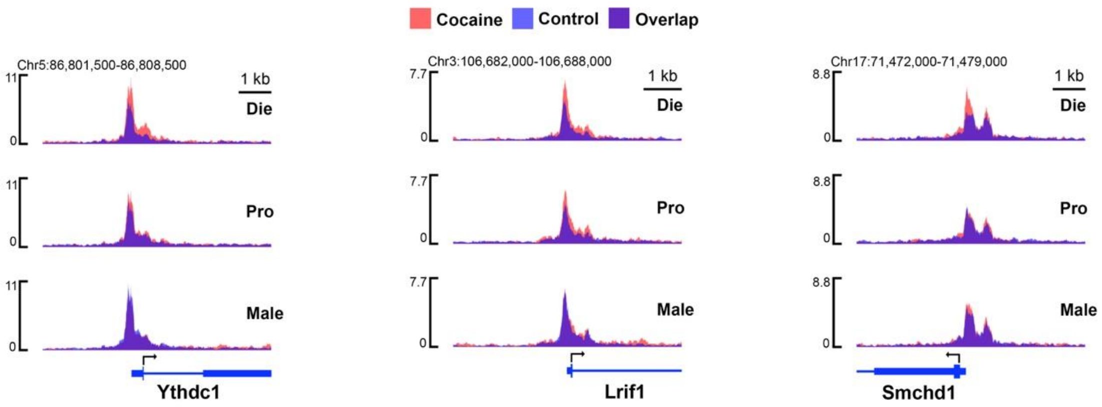

Supplementary Figure 14. Promoter regions of autosomal genes involved in X-inactivation are more accessible after cocaine in diestrus females. SparK plots of group-average normalized ATAC-seq reads (n = 2-3 replicates or 6-9 animals/group) are shown for autosomal genes involved in X-inactivation, whose expression is also altered in females by early-life stress (**Suppl. Figure 7A**), including regions overlapping the transcription start sites (TSSs) of *Ythdc1* (left), *Lrif1* (middle), and *Smchd1* (right), all of which are more accessible after acute cocaine specifically in diestrus females. Die, diestrus; Pro, proestrus; Male, males.

Supplementary Figure 15

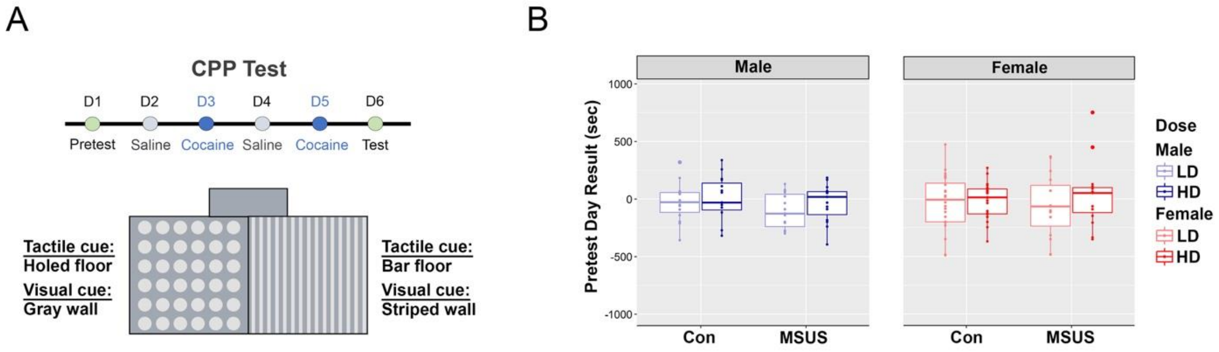

Supplementary Figure 15. Cocaine conditioned place preference (CPP) paradigm. (**A**) A schematic showing the timeline of the cocaine CPP test, which includes a pretest day, four conditioning days alternating between saline and cocaine treatment, and a test day (top), as well as an illustration of the CPP apparatus which has two chambers separated by a closeable corridor which are distinguishable by visual and tactile cues (bottom). (**B**) Plots showing the time spent in each compartment of the CPP apparatus by all animals across sex, group, and dose during the pretest day, demonstrating that animals in the experiment had no overall preference for either compartment. Box plots (box, 1st–3rd quartile; horizontal line, median; whiskers, 1.5× IQR). D, Day; Con, control group; MSUS, maternal separation combined with unpredictable maternal stress group; LD, low-dose; HD, high-dose.

Supplementary Figure 16

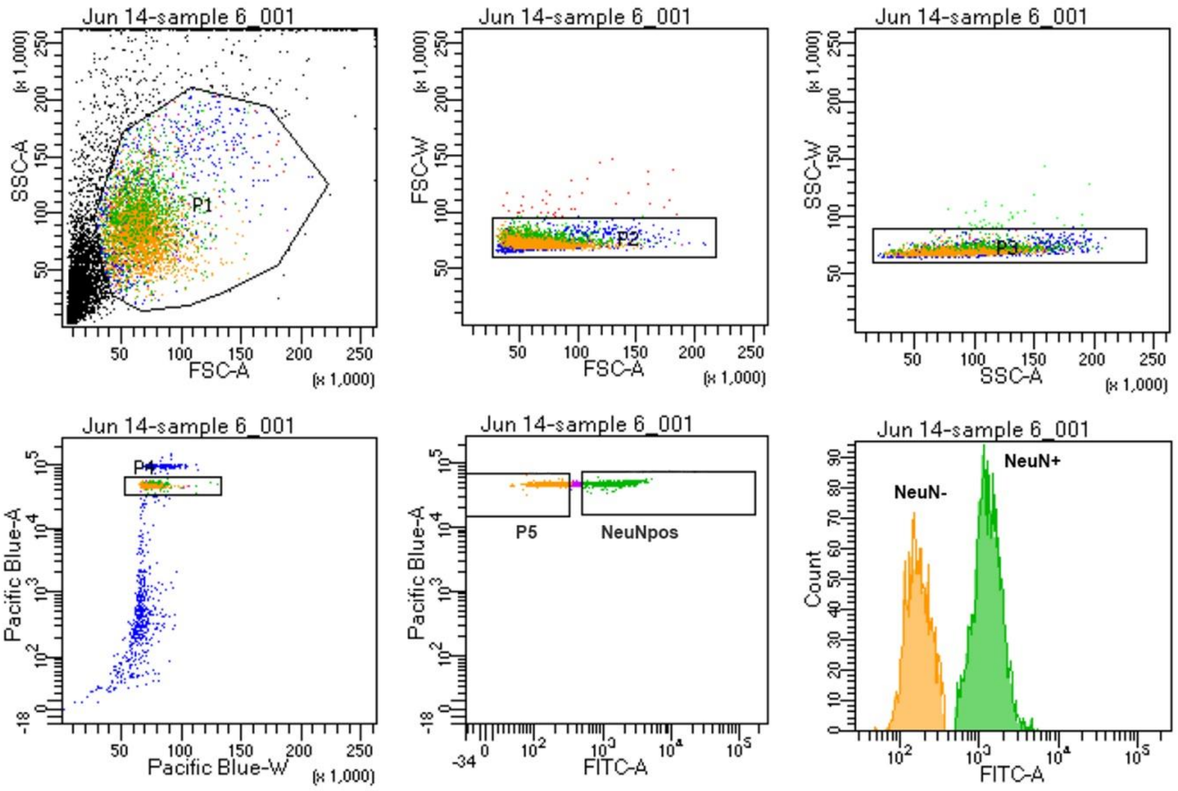

Tube: sample 6_001

Population	#Events	%Parent	%Total
All Events	10,000	####	100.0
P1	4,680	46.8	46.8
P2	4,642	99.2	46.4
P3	4,608	99.3	46.1
P4	3,871	84.0	38.7
P5	1,531	39.6	15.3
NeuNpos	2,212	57.1	22.1

Supplementary Figure 16. Purification of neuronal (NeuN+) nuclei with fluorescence-activated nuclei sorting (FANS). A representative FANS report demonstrating the gating procedure that allowed: 1) the separation of nuclei from cellular debris (P1-P3); 2) the separation of single, intact nuclei using the DAPI signal (P4); and 3) a specific purification of neuronal nuclei with a NeuN+ signal (P6) from non-neuronal (NeuN-) nuclei (P5).
